# Pathogenic adaptations of *Colletotrichum* fungi revealed by genome wide gene family evolutionary analyses

**DOI:** 10.1371/journal.pone.0196303

**Published:** 2018-04-24

**Authors:** Xiaofei Liang, Bo Wang, Qiuyue Dong, Lingnan Li, Jeffrey A. Rollins, Rong Zhang, Guangyu Sun

**Affiliations:** 1 State Key Laboratory of Crop Stress Biology in Arid Areas and College of Plant Protection, Northwest A&F University, Yangling, Shaanxi Province, China; 2 Department of Plant Pathology, University of Florida, Gainesville, United States of America; Universita degli Studi di Pisa, ITALY

## Abstract

The fungal genus *Colletotrichum* contains hemibiotrophic phytopathogens being highly variable in host and tissue specificities. We sequenced a *C*. *fructicola* genome (1104–7) derived from an isolate of apple in China and compared it with the reference genome (Nara_gc5) derived from an isolate of strawberry in Japan. Mauve alignment and BlastN search identified 0.62 Mb lineage-specific (LS) genomic regions in 1104–7 with a length criterion of 10 kb. Genes located within LS regions evolved more dynamically, and a strongly elevated proportion of genes were closely related to non-*Colletotrichum* sequences. Two LS regions, containing nine genes in total, showed features of fungus-to-fungus horizontal transfer supported by both gene order collinearity and gene phylogeny patterns. We further compared the gene content variations among 13 *Colletotrichum* and 11 non-*Colletotrichum* genomes by gene function annotation, OrthoMCL grouping and CAFE analysis. The results provided a global evolutionary picture of *Colletotrichum* gene families, and identified a number of strong duplication/loss events at key phylogenetic nodes, such as the contraction of the detoxification-related RTA1 family in the monocot-specializing graminicola complex and the expansions of several ammonia production-related families in the fruit-infecting gloeosporioides complex. We have also identified the acquirement of a RbsD/FucU fucose transporter from bacterium by the *Colletotrichum* ancestor. In sum, this study summarized the pathogenic evolutionary features of *Colletotrichum* fungi at multiple taxonomic levels and highlights the concept that the pathogenic successes of *Colletotrichum* fungi require shared as well as lineage-specific virulence factors.

## Introduction

The *Colletotrichum* genus is genetically diverse, comprising over 100 Ascomycota fungal species grouped into 10 major species complexes or species sensu lato [1, 2]. *Colletotrichum* species are also overwhelmingly successful phytopathogens, causing anthracnose foliar blight or fruit/stem rot on more than 3,000 plant species [3], and generating large economic losses on crops, vegetables, and fruit trees worldwide. While many *Colletotrichum* species are phytopathogenic, some interact with plants as endophytes, live freely as saprobes, or exhibit more than one lifestyle. In rare cases, *Colletotrichum* spp have been known to cause opportunistic animal infections [4, 5].

Most *Colletotrichum* pathogens penetrate the plant cuticle using melanized appressoria. Upon penetration, they differentiate infectious hyphae which spread intercellularly and/or intracellularly, and pass through biotrophic and necrotrophic infection phases sequentially [6]. Host interaction style varies among pathogen species, host organ/tissue types and plant developmental stages [7]. For instance, the biotrophic phase of *C*. *higginsianum* is limited to the first invaded epidermal cell whereas that of *C*. *graminicola* is present both at the advanced lesion margin and in the central colonization areas [3]. Species belonging to the gloeosporioides and actutaum complexes cause post-harvest fruit rots, in which the pathogens actively penetrate young fruit, persist quiescently for months, and reinitiate colonization when fruit begin to mature [8]. Host senescence or wounding can trigger the switch from quiescent endophyte to pathogenic colonizer [9–11].

Given the diverse taxonomic lineages and plant-interaction styles, it is difficult to assign a genus-wide representative pathogen model for study purposes, as knowledge gained from one pathosystem may not be directly transferred to another [7]. Yet, these variations may manifest through a unified mechanistic principle, where host defense levels and pathogen ‘stealth’ strategies together shape the interaction type (endotroph, biotroph, or necrotroph) and the time points at which phase shifts occur [7]. The entire genus may share both conserved and novel virulence factors tailored in lineage-specific manners for host/tissue adaptation. Identifying these virulence factors and characterizing their evolution are critical for *Colletotrichum* disease control and for better understanding the fundamental mechanisms of host-pathogen interaction. Comparative genomics thorough genome sampling both in and outside of the genus is an approach with high potential to identify virulence factors.

Currently, a dozen *Colletotrichum* genomes are publicly available [3, 12–18]. These representatives of the genus belong to six independent species complexes encompassing different plant-interaction styles (including endophytes, monocot and dicot foliar pathogens, and fruit pathogens). These genomes have been analyzed either separately or in combination to identify genomic features associated with host-adaptive evolutions [15–17], which concordantly reveal that *Colletotrichum* species may tailor their plant cell wall degrading enzymes (PCWDEs) and proteinases in accordance with their own infection styles. Thus the contents of these genomes are more likely to be grouped based on host range similarity rather than phylogenetic relatedness [15, 16]. *Colletotrichum* genomes are also known to be enriched with enzymes catalyzing secondary metabolite biosynthesis, many of which show phase-specific expressions during infection [3].

*Colletotrichum fructicola* is a recently established species belonging to the economically important gloeosporioides species complex. It is globally distributed and has a very broad host range, including over 50 plant species distributed in eight different families [7]. Diseases caused by *C*. *fructicola* are important economic concerns on many crops such as strawberry, apple, pear, and oil tea. On apple, natural *C*. *fructicola* isolates show pathogenic variation related to tissue/cultivar specificities [19], indicating that this broad host range species is made up of individual host-limited forms. A *C*. *fructicola* strain isolated from strawberry, Nara-gc5, has been genome sequenced [13], providing a reference for gene function studies and genome comparison purposes.

In this study, we sequenced a *C*. *fructicola* strain isolated from an apple Glomerellla leaf spot lesion in China and performed gene content comparison encompassing 13 *Colletotrichum* and 11 non-*Colletotrichum* genomes. The objectives of this study were several fold: first, by comparing representative *Colletotrichum* genomes with non-*Colletotrichum* genomes, we expected to identify genomic features conserved across the entire *Colletotrichum* genus, e.g. gene functions being genus-specific or expanded prior to the genus divergence; second, by characterizing gene content variation of different *Colletotrichum* species complexes, we expected to identify factors related to host adaptations among distinctive *Colletotrichum* lineages; third, to compare the intraspecific gene content variation between the two *C*. *fructicola* genomes derived from isolates of different hosts.

## Materials and methods

### Fungal isolate, sequencing, assembling and annotations

The *C*. *fructicola* 1104–7 isolate was obtained from an apple Glomerella leaf spot lesion in a private orchard in Hebei Province, China. Its *C*. *fructicola* species identity was confirmed by multi-locus concatenation phylogeny. The leaf sample was collected with the permission of the orchard owner. Pathogenicity test demonstrated that the isolate could cause apple bitter rot (ABR) and Glomerella leaf spot (GLS). The isolate was self-fertile and produced the *Glomerella* teleomorph in culture. Its morphological characteristics and sexual behavior fit the ‘plus’ strain descriptions [20, 21]. The isolate was cultured on potato dextrose agar and preserved as a 15% glycerol conidial stock at -80°C, and was deposited in the Agricultural Culture Collection of China (ACCC) under the accession number ACCC39328. Genomic DNA was extracted with freshly-collected mycelia from a 4-day potato dextrose broth shake culture (150 rpm, room temperature) based on a modified cetyl trimethylammonium bromide (CTAB) procedure [22]. Genome sequencing was performed with an Illumina HiSeq 2000 platform at the Novogene Genomic Sequencing Center, Beijing, China. The mean insertion size of sequencing libraries was 350 bp and the sequencing strategy was 100-bp pair-ends. Raw reads were trimmed with an in house perl script to remove low quality reads (N > 10%, or sQ ≤ 5) and reads with adaptor contamination. Clean reads were then *de novo* assembled using the AbySS assembler version 1.3.5 [23], with a Kmer value of 50. GapFiller version 2.0 [24] was used to further fill gaps and generate scaffolds. The generated genome assembly was deposited at GenBank under accession no. MVNS00000000.

Repetitive DNA elements were predicted with a combination of RepeatMasker version 4.0.5 and RepeatModeler version 1.0.8. To predict gene structures, Augustus version 3.1 [25], SNAP version 2013-11-29 [26], GeneMark-ES version 2.3c [27], and MAKER2 version 2.31.8 [28] were used in combination. Augustus and SNAP were trained with gene models of the JGI *Glomerella cingulate* 23 strain (http://genome.jgi.doe.gov/programs/fungi/index.jsf), GeneMark-ES was self-trained. Prediction results of Augustus, GeneMark-ES, and protein models of *G*. *cingulate* 23 were combined for a final MAKER2 integration. Predicted genes were functionally annotated with the Blast2GO software [29], putative functions were assigned based on BLASTP search against a local NCBI nr database (release date: 2016-09-01). Predicted transcript sequences and gene annotations were deposited as supplemental information. BUSCO version 1.2 [30] was used to evaluate the completeness of genome assembly and gene predictions. Genome alignment of 1104–7 and Nara_gc5 was performed with Mauve software version 2.4.0 [31], and single nucleotide polymorphism sites (SNPs) were extracted with the SNP-sites software [32].

### Phylogenomic analysis and gene family evolution

Predicted proteins encoded by a total of 24 fungal genomes (accessions listed in Table A in S1 File) were filtered (removing those containing less than 70 aa), and clustered into orthologous groups (Table B in S2 File) by OrthoMCL version 2.0.9 with an inflation value of 1.5 [33]. Single copy ortholog groups were then extracted for phylogenomic tree construction. Independent ortholog groups were aligned with MAFFT version 7 (http://mafft.cbrc.jp/alignment/server) and the conserved sites were extracted and concatenated with Gblocks version 0.91b [34]. Based on the concatenated dataset, a maximum-likelihood (ML) phylogenetic tree was constructed with RAxML version 8.1.1 [35] using the LG+G+I model chosen by ProtTest version 3.4 [36] with the bootstrap value set as 1,000.

Based on the ML dendrogram generated above, a calibrated species tree was constructed with the r8s software version 1.7 [37], analyses were based on penalized likelihood method and the TN algorithm. The *Colletotrichum* crown, Sordariomycetes crown, and Sordariomycetes-Leotiomycetes crown were chosen as calibration points [17, 38], predictions from a combination of four calibration schemes and three smoothing factors were compared to estimate divergence ranges. CAFE program version 3.1 was used for gene family expansion/contraction analysis [39], a universal lamda value (maximum likelihood value of the birth & death parameter) was assumed, and the best value was obtained by iterative calculations. Families showing significant size variance were identified based on 1,000 random samples and a p-value cutoff of 0.01, deviated branches were further identified based on the Viterbi algorithm in CAFE with a p-value cutoff of 0.05.

### Gene function predictions

Putative protein domains were identified by querying against a local Interproscan database (Jones et al. 2014). SMURF (http://jcvi.org/smurf/index.php) was used to predict putative secondary metabolite genes and clusters with the default parameters except that terpene cyclases (TCs) were identified by Hmmscan in HMMer version 3.0 [40] using the PFAM domain PF03936 (e-value, 1E-03). Candidate transcription factors (TFs) were identified with Hmmscan based on reported TF domains [41] with a cut-off e-value of 1E-03. Candidate cytochrome P450s (P450s) were identified by Hmmscan with PFAM domain PF00067 (cut-off e-value, 1E-03), and further classified into families and subfamilies following BLASTP against all named fungal CYPs (http://blast.uthsc.edu/). For family/subfamily assignment, the international cytochrome P450 nomenclature criteria were followed (i.e. P450s showing >40% identity were assigned to the same family) [42]. Candidate transporters were identified based on the TransportTP server (http://bioinfo3.noble.org/transporter/) with an e-value threshold of 1E-05. Candidate *Colletotrichum-*genus-specific genes were identified by BLASTP search against a local NCBI fungal database excluding *Colletotrichum* sequences (cut-off e-value, 1E-05).

Secretomes were identified using a procedure similar to that previously reported [43], in which SignalP version 4.1 [44], TMHMM Server version 2.0 [45], GPI-SOM [46] and WoLF PSORT [47] were run sequentially. Putative proteases were identified and classified by BLASTP querying against the MEROPS database (http://merops.sanger.ac.uk/) with a cut-off e-value of 1E-04, sequences containing mutated active sites or incomplete domains were removed. Carbohydrate utilizing enzymes were identified and classified based on BLASTP search against carbohydrate-active enzyme (CAZY) database (www.cazy.org) with a cut-off e-value of 1E-03. Functional enrichment tests were performed with FUNRICH version 2.1.2 [48].

## Results

### General features of the *Colletotrichum fructicola* 1104–7 genome

In total, 5.8 Gb pair-ended clean reads were assembled into 686 scaffolds with a total length of 57.1 Mb. The assembly size was similar to other *Colletotrichum* genomes such as the *C*. *fructicola* Nara_gc5 strain (55.6 Mb), *C*. *gloeosporioides* (53.2 Mb), *C*. *graminicola* (57.4 Mb) and *C*. *higginiasum* (53.4 Mb). The longest scaffold was 1.8 Mb and the N_50_ length was 339 kb. The average GC content was 53.2% and approximately 2.7% of the assembly consisted of repeat elements. Based on Mauve progressive alignment (Min LCB Weight = 250, Match Seed Weight = 15), 95.01% (54.3 Mb) of the 1104–7 genome could be aligned with Nara_gc5 (length > 500 bp), among which 50.2% (27.2 Mb) were in blocks longer than 100 kb and 97.4% (52.9 Mb) were in blocks longer than 10 kb, the aligned sequences shared 98.7% nucleotide identity and the average SNP frequency was 0.26%.

An integrative *ab-initio* approach predicted 17,827 protein-encoding genes. Among the 17,827 putative proteins, 92.46% (16,483) had at least one BLASTP hit in a local NCBI non-redundant (nr) database (e-value cut-off 1E-05), 52.9% (9,430), 64.3% (11,457), 58.1% (10,349), and 72.8% (12,973) could be annotated based on Gene Ontology (GO), Clusters of Orthologous Groups (COG), Kyoto Encyclopedia of Genes and Genomes (KEGGs) and PFAM respectively. In BUSCO analysis, 96.8% of the fungal core genes had hits as ‘complete’ and 90.5% had hits as ‘complete and single-copy’, demonstrating completeness of the annotation. Based on an independent project (Liang et al., unpublished), 84.8% (15,114) of the 1104–7 predicted genes contained at least five RNA-seq reads among a total of ~65 million tags (sequenced samples included conidia, *in vitro* appressoria, cellophane infectious hyphae, and infected plant).

### Gene content variation between the two *Colletotrichum fructicola* genomes

The 1104–7 genome was compared with the other publicly available *C*. *fructicola* genome, Nara_gc5 (GenBank accession: ANPB00000000.1). To minimize annotation pipeline-related variation, the Nara_gc5 assembly was re-annotated with the same parameters as 1104–7, 17,844 gene models were predicted in total.

Based on OrthoMCL clustering of the two genomes, 980 genes were specific to 1104–7 (unclustered or clustered only with proteins from the same genome), among which 65.3% (640) had RNA-seq evidence support (Liang et al., unpublished data), 616 (62.9%) had significant NCBI nr BlastP hit (e-value cut-off 1E-05) and 146 (14.9%) contained PFAM domains. Top enriched PFAM functions were related to DNA transposition (hAT family protein, gag, Tc5 transposase), apoptosis (caspase, NACHT), DNA binding (helix-turn-helix, zinc knuckle), protein-protein interaction (ankyrin repeats), binding (ferritin-like, CFEM) and aspartyl protease (Table 1). 1,128 genes were specific to Naga_gc5, 708 (62.8%) had significant BlastP hits and 286 (25.4%) contained PFAM domains, top enriched functions were related to heterokaryon incompatibility, DNA transposition (DDE endonuclease; MULE transposase), protein kinase and patatin-like phospholipase activities.

**Table 1:** Top enriched PFAM domains in OrthoMCL-defined isolate-specific genes in *C*. *fructicola* 1104–7 and Nara-gc5.

PFAM	Annotation	Number	Fold Enrichment	B-H *P*-value^a^
1104–7				
PF05699	HAT family C-terminal dimerisation	9	49	7.6E-14
PF03732	Retrotransposon gag protein	8	44	1.7E-11
PF00656	Caspase domain	8	37	2.0E-10
PF05225	Helix-turn-helix, Psq domain	8	34	4.4E-10
PF00098	Zinc knuckle	7	25	2.2E-07
PF13646	HEAT repeats	6	29	6.9E-07
PF12796	Ankyrin repeats (3 copies)	17	5	1.4E-06
PF03221	Tc5 transposase DNA-binding domain	5	25	2.5E-05
PF13650	Aspartyl protease	4	35	3.9E-05
PF13668	Ferritin-like domain	4	25	0.0003
PF05730	CFEM domain	6	5	0.002
PF05729	NACHT domain	8	11	0.008
Nara_gc5				
PF11702	Protein of unknown function (DUF3295)	3	32	0.006
PF06985	Heterokaryon incompatibility protein	17	3	0.007
PF10551	MULE transposase domain	2	40	0.02
PF01734	Patatin-like phospholipase	4	15	0.02
PF13358	DDE superfamily endonuclease	2	40	0.03
PF00069	Protein kinase domain	11	3	0.03

^a^B-H: Benjamini-Hochberg adjusted

**Fig 1:**
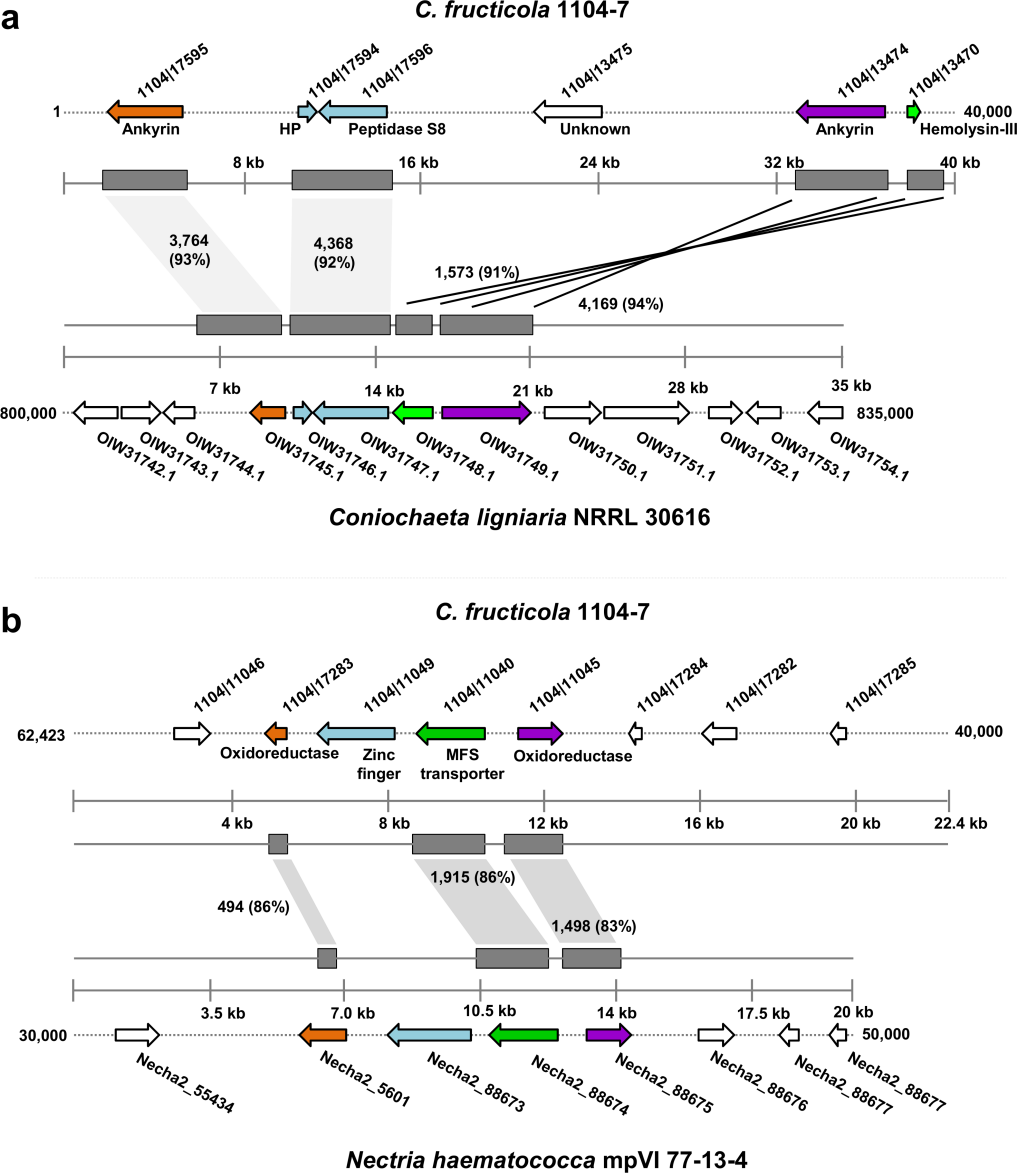
The two putatively fungus-to-fungus horizontally-transferred gene clusters present in the lineage-specific regions of the 1104–7 genome. Syntenic DNA blocks (identified based on Blast search) are in dark grey boxes, genes are in arrowheads, orthologous genes are in the same color, alignment length and nucleotide percentage identity (in bracket) are also shown. Maximum likelihood based phylogenetic trees of the HGT genes are shown in S1 Fig.

Fungal lineage-specific (LS) genomic regions are often enriched with genes mediating host interactions and niche adaptations, we therefore identified and analyzed LS regions in 1104–7 and Nara_gc5. Long (> 10 kb) and unaligned DNA blocks were identified by performing Mauve alignment, their lineage specificities were further confirmed by genome BlastN search. In total, 0.62 Mb LS regions were identified in 1104–7 (distributed on 32 contigs, containing 118 genes), 0.33 Mb LS regions were identified in Nara_gc5 (distributed on 20 contigs, containing 72 genes). In 1104–7 and Nara_gc5, 61.9% (73) and 39.4% (28) isolate-specific proteins had significant BlastP hits in a local NCBI non-redundant (nr) database (e-value cut-off 1E-05, coverage > 50%), the ratios were much lower compared with the genome backgrounds (approximately 90% for both). Interestingly, in 1104–7 and Nara_gc5, 21.1% (25) and 8.4% (6) of isolate-specific genes respectively, had only non-*Colletotrichum* homologs (BLASTP e-value cut-off 1E-05, query coverage > 50%), or were more similar to non-*Colletotrichum* sequences than to *Colletotrichum* sequences (BLASTP e-values for best hits differed by at least 1E+10 fold). The frequencies of genes with such characteristics were only 1% in control groups made up of randomly-selected genes (Table 2, type III + type IV). As a comparison, the frequencies of genes having only *Colletotrichum* hits (Table 2, type II) were similar between LS and control groups in both 1104–7 (9% vs. 12.7%) and Nara_gc5 (10% vs 15.5%). Thus, genes located within LS regions evolve more dynamically, and a strongly elevated proportion of genes are closely related to non-*Colletotrichum* sequences. Phylogenetically, many non-*Colletotrichum* related LS genes were deeply-rooted with poor bootstrap support, making it difficult to infer gene evolutionary histories (data not shown). However, two putative fungus-to-fungus horizontal transfer events (HGTs), involving nine LS genes in total, were identified among non-*Colletotrichum* related genes in the 1104–7 genome. The two HGTs were supported by both gene order collinearity (Fig 1) and gene phylogeny patterns (S1 Fig). The first HGT cluster contained five genes, among which were two ankyrin proteins, one serine peptidase, one hemolysin-III domain protein, and one hypothetical protein, the cluster genes were most closely related to genes from *Nectria haematococca* and *Coniochaeta ligniaria*, and the gene orders among the three species were collinear, nucleotide identities for aligned DNA blocks reached over 90%. The second HGT cluster probably functions in secondary metabolism as it contained two oxidoreductases, one MFS transporter, and one zinc finger transcription factor. The cluster genes were most closely related to genes from *N*. *haematococca* and the two gene clusters were collinear, nucleotide identities for aligned DNA blocks were over 80%.

**Table 2:** BlastP hit characteristics of randomly-chosen genes and genes located in lineage-specific (LS) genomic regions against the NCBI nr database.

	1104–7 Ref^1^	1104–7 LS^2^	Nara_gc5 Ref	Nara_gc5 LS
Type I^3^	160 (80%^8^)	33 (28%)	160 (80%)	11 (15.5%)
Type II^4^	18 (9%)	15 (12.7%)	20 (10%)	11 (15.5%)
Type III^5^	1 (0.5%)	22 (18.6%)	1 (0.5%)	4 (5.6%)
Type IV^6^	1 (0.5%)	3 (2.5%)	1 (0.5%)	2 (2.8%)
Type V^7^	20 (10%)	45 (38%)	18 (9%)	43 (61%)
Total	200	118	200	71

^1^Ref, genes randomly chosen from the genome

^2^LS, genes located in lineage-specific (LS) regions

^3^Type I, conserved genes having significant BlastP hits (e-value cut-off 1E-05, query coverage > 50%) both in and out of the *Colletotrichum* genus; e-value ratios for best BlastP hits (E_in_/E_out_) ≤ 1E+10.

^4^Type II, genes having significant BlastP hits only in the *Colletotrichum* genus.

^5^Type III, genes having significant BlastP hits only outside of the *Colletotrichum* genus.

^6^Type IV, genes having better BlastP hit outside of the *Colletotrichum* genus (E_in_/E_out_ > 1E+10).

^7^Type V, no BlastP hit found.

^8^%, Relative percentage.

### Divergences and overall gene gain and loss patterns among *Colletotrichum* lineages

**Fig 2:**
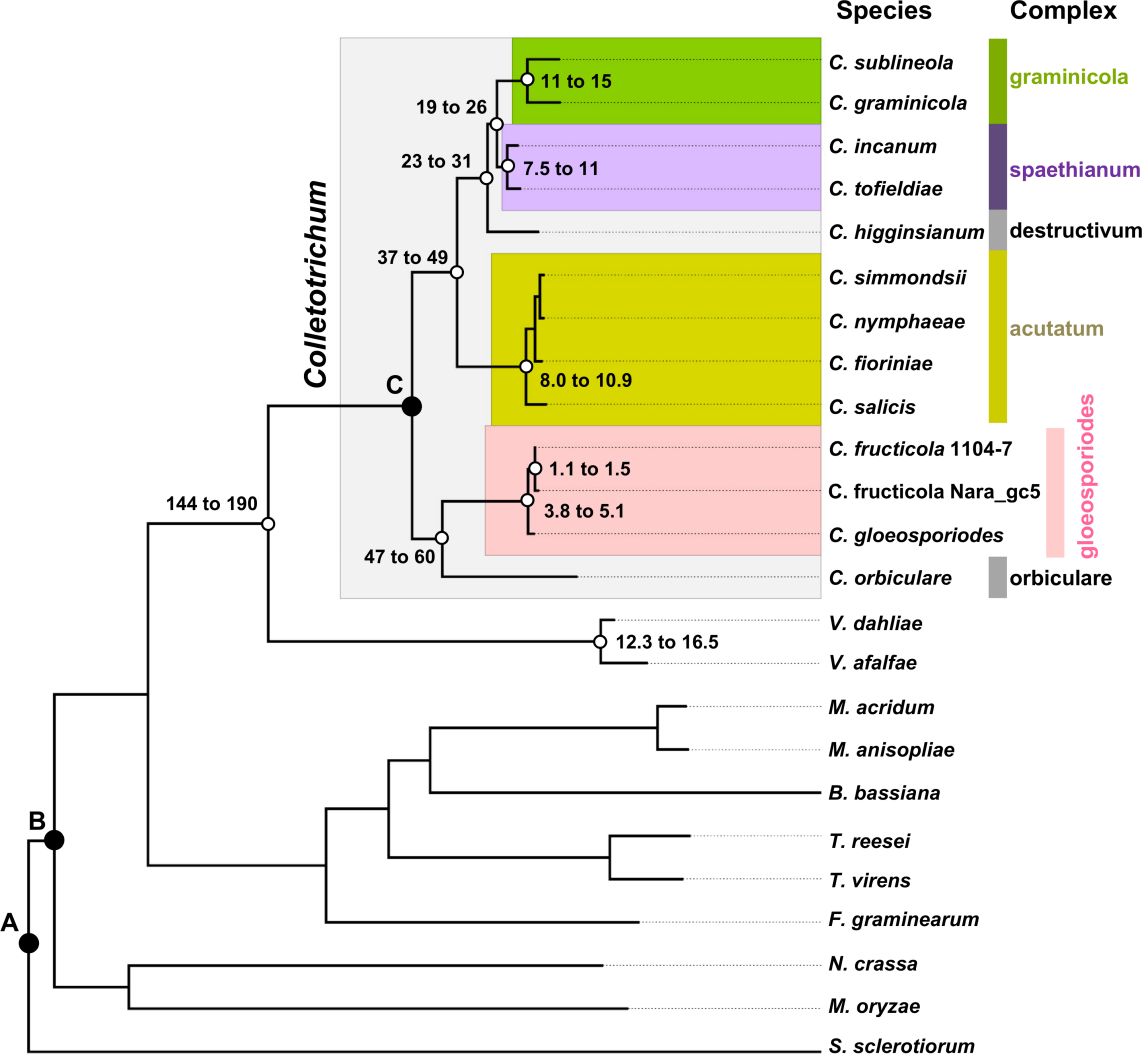
Maximun-likelihood phylogenetic tree constructed from 1,212 single-copy core genes and divergence time estimation using r8s analysis. A, B and C are calibration points, divergence times are shown in million years, the ranges were calculated based on estimations with different combinations of smoothing factors and calibration schemes (see Table C in S2 File for detail).

OrthoMCL clustering identified 1,212 core single-copy ortholog groups among the 24 compared *Colletotrichum* and non-*Colletotrichum* genomes. A maximum-likelihood (ML) phylogenomic tree was constructed based on their concatenated alignment. On the ML tree, all branches received 100% bootstrap value support. Lineage divergence times were then estimated in r8s, for which the combined effects of three smoothing factors (1, 100, 1,000), and four calibration schemes were tested (Table C in S2 File), the results were presented in Fig 2 and Table C in S2 File. The two *C*. *fructicola* strains, 1104–7 and Nara_gc5, diverged approximately 1.3 million years (My) ago whereas *C*. *fructicola* and *C*. *gloeosporioides* diverged approximately 4.5 My ago. The gloeosporioides complex includes two phylogenetic clades, Musae and Kahawae [49], the fact that both *C*. *fructicola* and *C*. *gloeosporioides* belong to the Musae clade precluded origin estimation for the gloeosporioides complex. Origins for the other three complexes (graminicola, spaethianum, and acutatum) were similar, ranging between 9.0 and 13 My ago. The gloeosporioides and acutatum complexes, two pathogen groups commonly associated with post-harvest fruit infections, diverged by at least 47 My (the shortest divergence estimation for gloeosporioides and orbiculare complexes).

**Fig 3:**
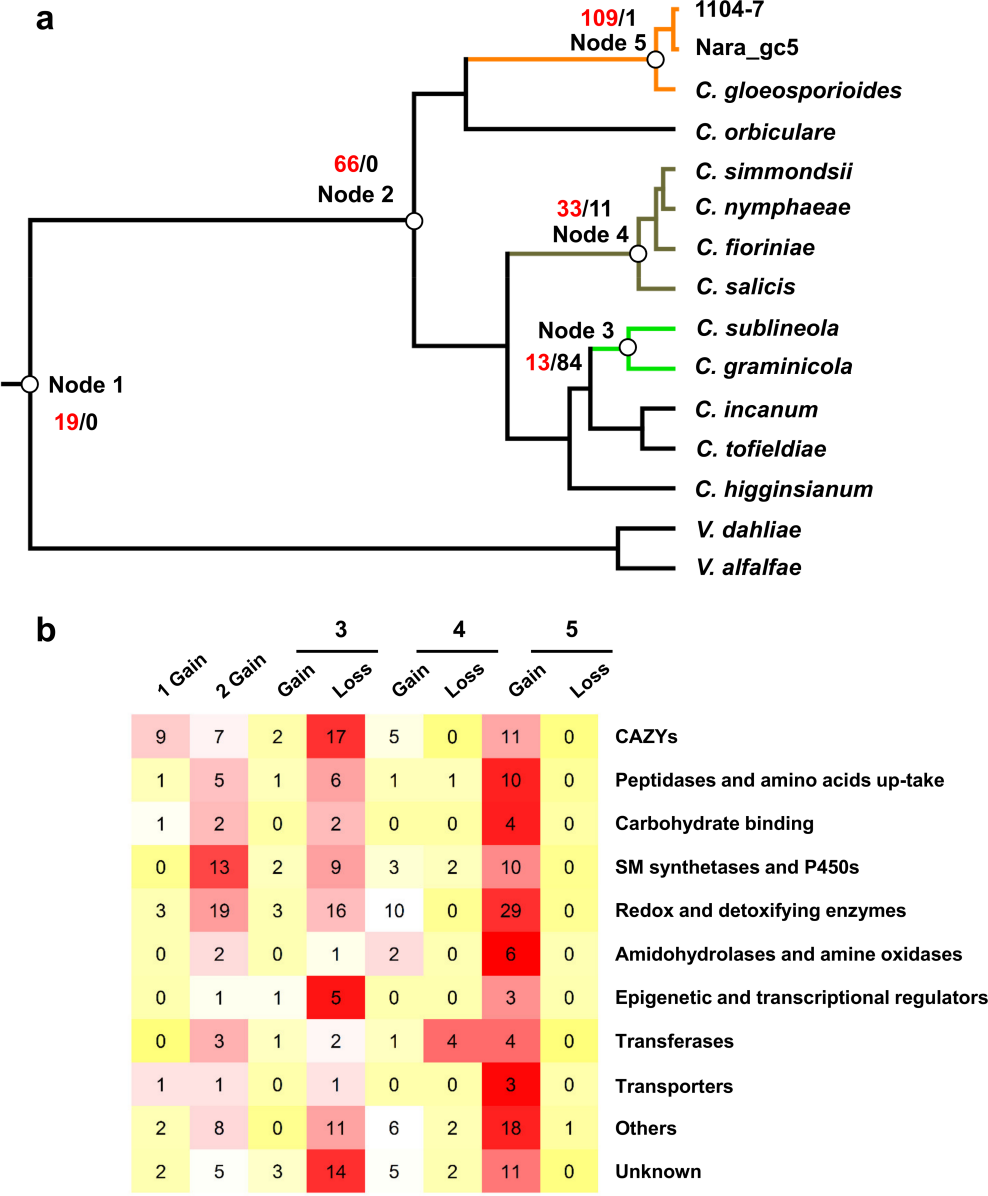
Gene gain and loss patterns at major five nodes of the *Colletotrichum* phylogeny. (**a**) Number of families significantly expanded (red) or contracted (CAFE analysis, family *P* < 0.01, Viterbi *P* < 0.05). (**b**) Functional categories of the families significantly expanded or contracted at indicated nodes.

CAZYs, secreted proteases, secondary metabolite synthetases, cytochrome P450s, transporters, and small secreted proteins (SSPs) are known virulence factors in fungi. Putative genes belonging to these functional categories were identified from the compared genomes via a custom prediction pipeline. In general, *Colletotrichum* genomes contained more virulence genes compared with non-*Colleotrichum* genomes (S2–S7 Figs), with the enrichments of CAZYs, cytochrome P450s, transporters, and SSPs being marked. From a total of 4,596 families (defined either based on PFAM domain or annotated functional category), CAFE based analysis of gene gain and loss patterns identified 454 families evolving in a non-random birth and death manner at a 0.01 family-wise significance level. For these families, the expected expansions/contractions and the corresponding Viterbi *p*-values were calculated for individual branches. Five branches closely related to *Colletotrichum* evolution were examined in greater detail (Fig 3A). These branches contained the most recent common ancestor (MRCA) of Glomerellales (node 1), the *Colletotrichum* MRCA (node 2), the graminicola complex MRCA (node 3), the *acutatum* complex MRCA (node 4), and the gloeosporioides complex MRCA (node 5). At a family-wide significance threshold of 0.05, 208 non-redundant families showed significant expansions/contractions (Table D in S2 File). The overall gene gain and loss patterns associated with these five nodes are shown in Fig 3. Consistent with previous reports [15, 16], GH43, AA7, and NLPs were strikingly expanded at the acutatum complex MRCA.

In general, the graminicola complex MRCA (node 3) was dominated by gene loss whereas the Glomerellales MRCA (node 1), the *Colletotrichum* MRCA (node 2), and the gloeosporioides complex MRCA (node 5) were dominated by gene gains (Fig 3A). A large number of gene families being expanded at the Glomerellales MRCA (node 1) were CAZYs, or more specifically ones related to pectin degradation. Other nodes were characterized by different expansion/contraction patterns with families experiencing significant size changes related to secondary metabolism, P450s, oxidoreductases, and detoxifications among others (Fig 3B).

### Gene family evolution prior to *Colletotrichum* and *Verticillium* divergence

At the Glomerellales MRCA (node 1), 19 families were significantly expanded (Viterbi *P* < 0.05). Interesting, many of these families were functionally related to degrading pectins (PL1, PL3, GH28, GH78, GH88, GH43, CBM67), celluloses or hemicelluloses (GH43, AA3, and AA9). Thus, the Glomerellales MRCA evolution involves a strong expansion of plant cell wall degrading enzymes (PCWDEs).

**Fig 4:**
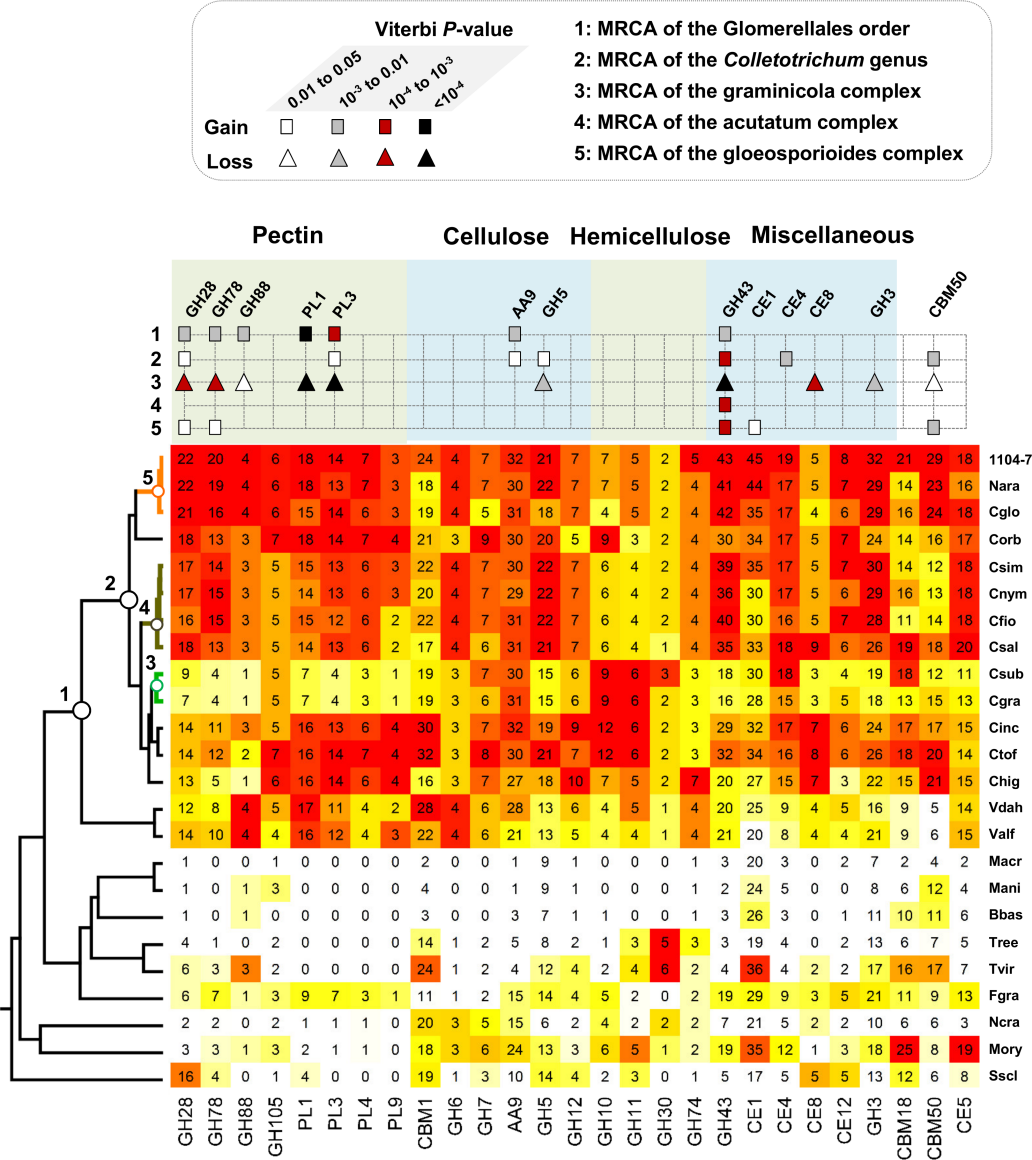
Content variation of CAZY families with plant cell wall degrading activity or known to be important for plant pathogen interactions. Species abbreviations: Nara, *Colletotrichum fructicola* Nara_gc5; Cglo, *C*. *gloeosporioides*; Corb, *C*. *orbiculare*; Csim, *C*. *simmondsii*; Cnym, *C*. *nymphaeae*; Cfio, *C*. *fioriniae*; Csal, *C*. *salicis*; Csub, *C*. *sublineola*; Cinc, *C*. *incanum*; Ctof, *C*. *tofieldiae*; Chig, *C*. *higginsianum*; Vdah, *Verticillium dahliae*; Valf, *V*. *alfalfae*; Macr, *Metarhizium acridum*; Mani, *M*. *anisopliae*; Bbas, *Beauveria bassiana*; Tree, *Trichoderma reesei*; Fgra, *Fusarium graminearum*; Ncra, *Neurospora crassa*; Mory, *Magnaporthe oryzae*; Sscl, *Sclerotinia sclerotiorum*.

*Colletotrichum* genomes are known to be enriched with PCWDEs [15, 16], we further examined major PCWDE-related CAZY families to gain a global insight into their evolutions (Fig 4). Gene family expansions were obvious with both the Glomerellales MRCA (node 1) and the *Colletotrichum* MRCA (node 2), each containing seven significantly expanded families, suggesting that the elevated PCWDE content in *Colletotrichum* was due to stepwise expansions. Within the *Colletotrichum* genus, the gloeosporioides complex showed obvious CAZY gains whereas the graminicola complex showed obvious CAZY losses, which were consistent with previous reports [15, 16].

### Gene family evolution at the *Colletotrichum* MRCA

**Fig 5:**
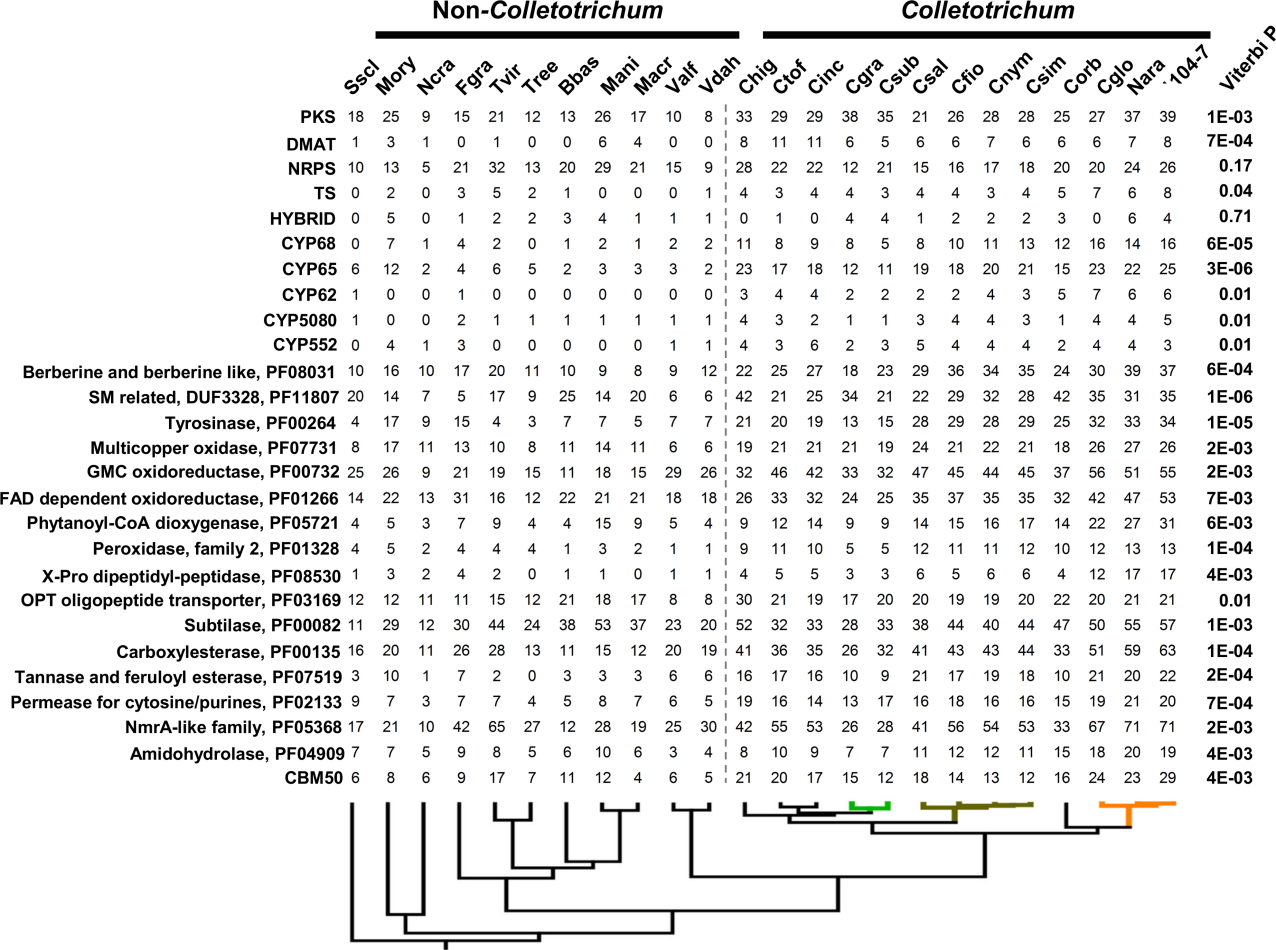
Copy number differences of selected gene families (defined based on PFAM or functional predictions) between non-*Colletotrichum* and *Colletotrichum* species. For each family, the Viterbi P value calculated with CAFE is shown on right.

At the *Colletotrichum* MRCA (node 2), 66 families were significantly expanded (Viterbi *P* < 0.05, Fig 5, Table D in S2 File). The most strongly-expanded family (Viterbi *P* = 1E-06) contained a PF11807 domain. While most PF11807 proteins are functionally unknown, the *Ustilaginoidea virens* ustYa and ustYb participate in the biosynthesis of the ribosomal peptide-derived toxin UstiloxinB [50], and the *Talaromyces islandicus* CctP functions in synthesizing the NRPS mycotoxin cyclochlorotine [51]. The second and fourth most strongly-expanded families were CYP68 and CYP65, two groups of cytochrome P450s being also related to secondary metabolite biosynthesis. CYP62, CYP5080, CYP552, as well as PKSs, and DMATs were also strongly expanded (*Viterbi P* < 0.01). Moreover, the expansion extent of berberine bridge enzymes (BBEs, PF08031), a family of flavin-dependent oxidoreductases critical for isoquinoline alkaloid biosynthesis [52, 53], ranked 11^th^ in expansion significance among all families. These results together supported a strong diversification in secondary metabolite production at the *Colletotrichum* MRCA.

Redox enzymes may contribute toward fungal pathogenesis in multiple ways, such as oxidative breakdown of cellulose and hemicellulose, synthesizing toxins, and counteracting plant-derived phenolic compounds. Tyrosinase (PF00264), type II peroxidase (PF01328), and GMC oxidoreductase (PF00732) were all strongly expanded at the *Colletotrichum* MRCA (Viterbi *P* < 0.01).

Protein families being strongly expanded at the *Colletotrichum* MRCA also included ones functioning in peptide degradation (e.g. x-pro dipeptidyl-peptidase, subtilase), nutrient uptake (e.g. OPT oligopeptide transporter, cytosine/purine permease), transcriptional regulation (e.g. NmrA-like protein), and chitin binding (e.g. CBM50) among others. Worthy to note, PF00135 (carboxylesterases) and PF07519 (tannase and feruloyl esterase activities), two detoxification-related families, were also strongly expanded (*Viterbi P* = 1E-04 and 2E-04 respectively). Carboxylesterase detoxifies xenobiotics (toxins or drugs) in animals [54]. Tannase degrades tannins, a group of plant defense related phenolic compounds [55] whereas feruloyl esterases facilitate xylan and pectin degradation [56].

OrthoMCL clustering identified three protein families showing *Colletotrichum* lineage-specific loss (present in all 11 compared non-*Colletotrichum* genomes, but none of the 13 *Colletotrichum* genomes). All three families were made up of single-copy orthologs, including one putative Ca^2+^/calmodulin-dependent protein kinase (CAMK, corresponding to XP_003717191 in *Magnaporthe oryzae*), one CofD_Yvck family protein (XP_003717966 in *M*. *oryzae*) and one lacking any function-indicative signature (XP_003715556 in *M*. *oryzae*). The CAMK gene lacks distinct ortholog in *S*. *cerevisiae* and no obvious phenotype was observed with the gene deletion mutant in *Fusarium graminearum* (FGSG_05549) [57]. CofD_Yvck family protein is related to carbon metabolism, but no fungal gene has been characterized.

### Genes families being specifically conserved among *Colletotrichum* genomes and *Colletotrichum* genus-specific SSPs

Based on OrthoMCL clustering, 260 families were identified to be *Colletotrichum*-specific among compared genomes and contained proteins from all 13 *Colletotrichum* genomes (Table E in S2 File). These genus core families contained members known or putatively important for plant infection, especially for appressorium functions, such as CAP22 [58], CAS1-like proteins [59], CFEMs [60], putative cutinase and ligninase. Four families were made of *Colletotrichum* genus-specific SSPs (defined by NCBI nr BLASTP, e-value cut-off 1E-05), which included the previously identified *C*. *higginsianum* effector candidates EC2 and EC65 [61], and one CFEM domain protein.

Based on queries of a local installation of the NCBI fungal database, we identified 939 *Colletotrichum* genus-specific SSPs. These proteins contained a predicted secretion signal, were less than 300 aa, and lacked a BlastP hit (E-value cutoff = 1E-05) in other fungal species. 29 genus-specific SSPs contained recognizable PFAM domains (eight domains in total, Table 3). PF14856 (Hce2) corresponds to the *Cladopsorium fulvum* Ecp2 effector which contains a necrosis-inducing activity [62]. PF05730 (CFEM) is functionally associated with fungal pathogenesis. PF08881 (CVNH), PF01822 (WSC), and PF00024 (PAN domain) are related to protein-oligosaccharide interactions. PF12296 (HsbA) and PF06766 (Hydrophobin2) are related to hydrophobic surface binding.

**Table 3:** PFAM domains contained by *Colletotrichum* small secreted proteins which lack significant BlasP hit (e-value cut-off 1E-05, query coverage > 50%) outside the genus.

PFAM ID	Annotation	Representative proteins^1^
PF12296	HsbA, hydrophobic surface binding protein A	EQB52112.1 (*C*. *gloeosporioides*, 1E-11) ENH89122.1 (*C*. *orbiculare*, 7E-11)
PF09792	Ubiquitin 3 binding protein But2 C-terminal domain	ENH78092.1 (*C*. *orbiculare*, 1.6E-05)
PF08881	CVNH domain	KDN63891.1 (*C*. *sublineola*, 2.5E-05) KDN62312.1 (*C*. *sublineola*, 8.7E-11) KZL65396.1 (*C*. *tofieldiae*, 3E-09)
PF14856	Hce2, putative necrosis-inducing factor	XP_007602516.1 (*C*. *fioriniae*, 3E-11) KZL66113.1 (*C*. *tofieldiae*, 2E-12) EQB50157.1 (*C*. *gloeosporioides*, 5E-13)
PF05730	CFEM	XP_007285601.1 (*C*. *fructicola*, 1.4E-09) ENH76065.1 (*C*. *orbiculare*, 1.2E-09)
PF01822	WSC, a putative carbohydrate binding domain	XP_007598049.1 (*C*. *fioriniae*, 1E-06) KXH31806.1 (*C*. *simmondsii*, 5.6E-08) KXH62552.1 (*C*. *nymphaeae*, 4.2E-08)
PF06766	Fungal hydrophobin	KZL63596.1 (*C*. *incanum*, 8.3E-05) ENH81598.1 (*C*. *orbiculare*, 6.6E-09)
PF00024	PAN domain	XP_007279807.1 (*C*. *fructicola*, 2.5E-05)

^1^Representative proteins, each GenBank accession is followed by a parenthesis showing the species name and PFAM domain hit E-value.

### Horizontal transfer of a RbsD/FucU fucose transporter from bacterium to the *Colletotrichum* ancestor

**Fig 6:**
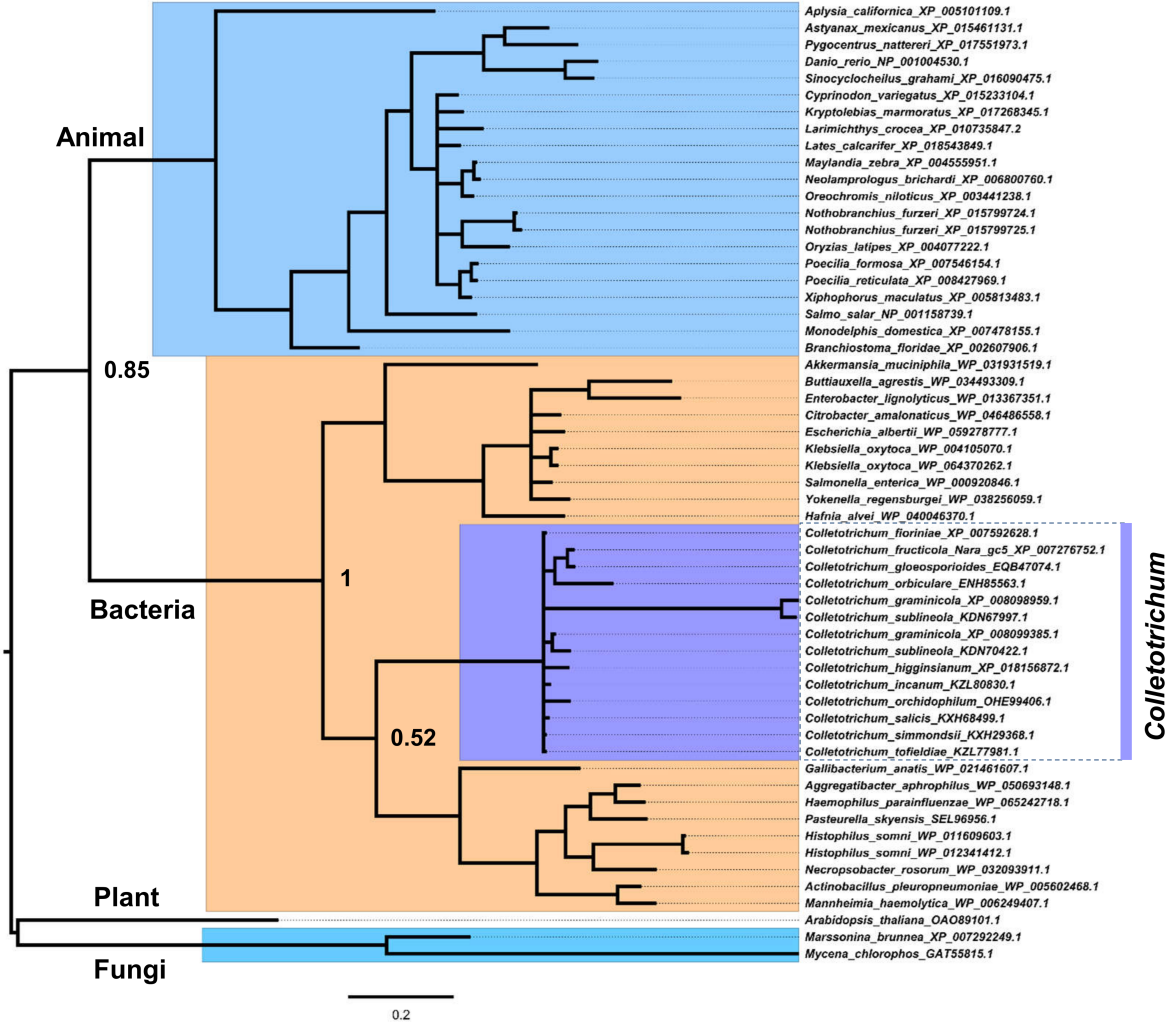
Putative bacteria-to-*Colletotrichum* horizontal transfer of the RbsD/FucU fucose transporter family (PF05025). Bayesian phylogenetic tree was constructed with the best fungal, bacterial, animal and plant BLASTP hits of the *Colletotrichum* RbsD/FucU fucose transporters in the NCBI nr database. The tree was constructed with MrBayes, WAG+G substitution model, 5 × 10^6^ mcmc generations, sample frequency = 1000, first 25% discarded as burn-in, numbers indicate posterior probabilities.

InterProScan search (cutoff E-value, 1e-04) and manual inspection identified tens of PFAM domains specific to the *Colletotrichum* genus among compared genomes and being present in more than one species. The co-occurrence of these domains in different genomes made it unlikely that their presence was due to DNA contaminations. BlastP searches showed that most protein homologous of these proteins distributed sporadically among fungi, or were specific to the *Colletotrichum* genus, making it hard to predict their evolutionary histories. However, one family, RbsD/FucU fucose transporter (PF05025), showed strong signatures of bacteria-to-fungi transfer. This RbsD/FucU fucose transporter (PF05025) family is conservatively present among all compared *Colletotrichum* genomes. In the NCBI nr database, the *Colletotrichum* proteins had homologs in diverse bacteria and animal species, but had no homolog across the fungal kingdom (BlastP, cutoff *P* = 1E-05). Phylogenetically, the *Colletotrichum* proteins formed a monophyletic clade nested within bacterial lineages with strong statistical support (Fig 6). Such combined patterns of taxonomic distribution and phylogenic topology supported bacteria-derived gene gain by the *Colletotrichum* ancestor. The genus-wide conservation of this family indicates its importance for lineage-specific adaptations. L-fucose is a major constituent of N-linked glycans, which distribute widely on the cell surfaces of microbes, plants and animals, L-fucose is also abundant in soil and can be used as the sole carbon source by several groups of microorganisms [63]. The acquisition of the RbsD/FucU fucose transporter may benefit *Colletotrichum* species in natural nutrient competition.

### Gene family evolution related to species complex diversification

**Fig 7:**
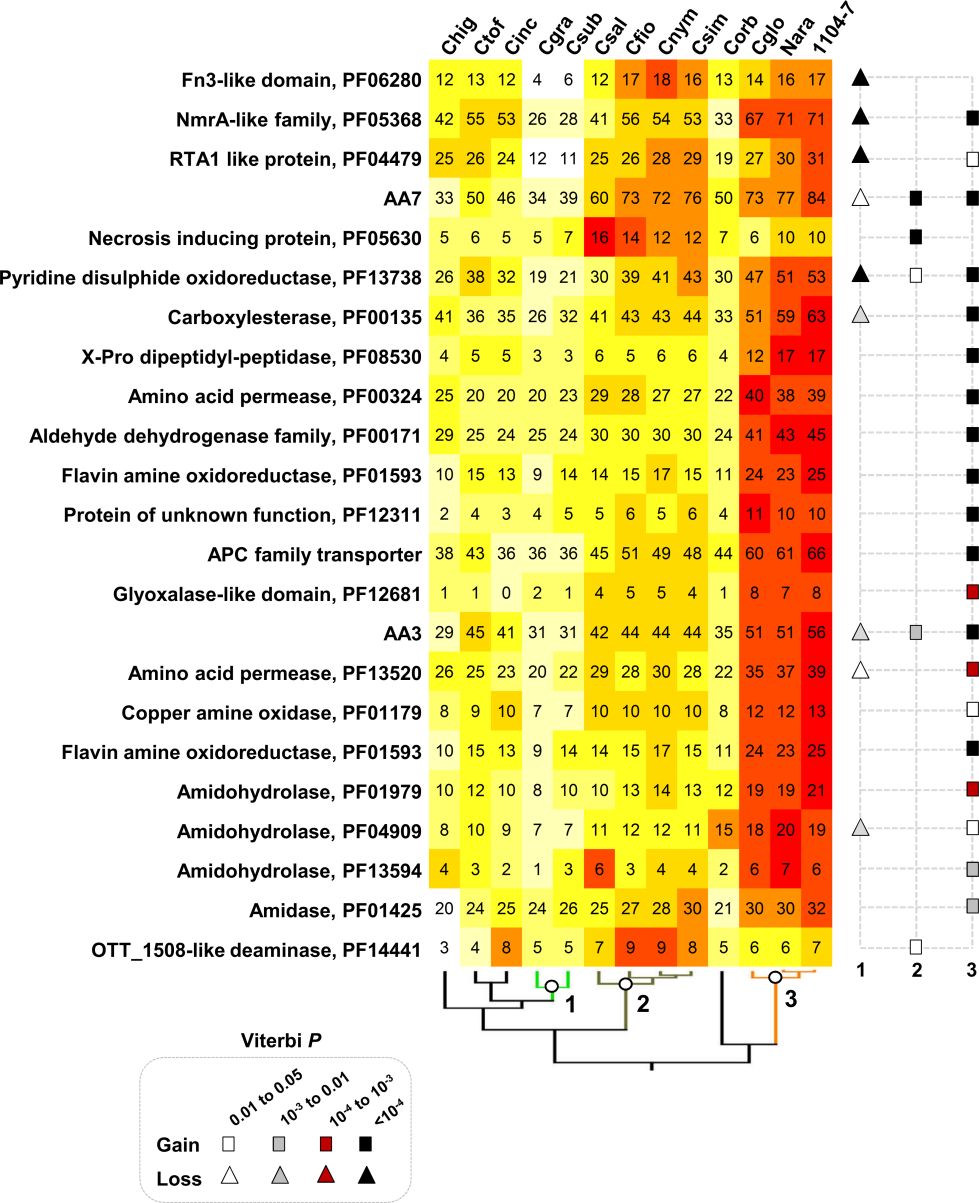
Evolution of gene families related to species complex diversification.

Species in the graminicola species complex contain a strongly reduced set of pectin-degrading enzymes associated with monocot host adaptation [3, 16]. In this study, we showed that a number of gene families functioning beyond pectin degradation were also reduced (Fig 3, Fig 7, Table D in S2 File). Among these families, Fn3-like protein (PF06280), NmrA-like protein (PF05368), and RTA1 (PF04479) showed the strongest reductions. The Fn3 domain is frequently found in streptococcal C5a peptidases (SCP) and adhesin/invasion proteins [64]. NmrA-like proteins are related to transcriptional regulation. The RTA1 protein family (PF04479) contains export proteins transporting antimicrobial compounds such as sphingoid bases and 7-aminocholesterol. Overexpression of RTA1 proteins confer drug or toxin resistance in yeast [65].

At the gloeosporioides species complex MRCA, the main functional categories of expanded gene families included redox and detoxifying enzymes, CAZYs, peptidases and amino acid transporters (Fig 3). The strong expansions observed with one putative peptidase family (PF08530) and two putative amino acid transporter families (PF00324, PF13520) indicated an improved capacity of the gloeosporioides complex to utilize protein-derived nutrients. Moreover, among the five non-redundant amidohydrolase families and two amine oxidase families catalyzing ammonia production, four amidohydrolase families (PF01979, PF13594, PF01425, PF04909) and two oxidase families (PF01593, PF01179) were significantly expanded (Viterbi *P* < 0.05), indicating an improved capacity to produce ammonia.

## Discussion

*Colletotrichum* species are genetically diverse and cause diseases on a wide range of plant species. Although differing considerably in host specificity and symptom appearance, most pathogens infect as hemibiotrophs, subverting host defense reactions first, and initiating host killing and host cell wall degradations thereafter. These phenomena support a universal infection strategy and perhaps underlying molecular mechanisms [6,7]. On the other hand, the considerable variation of plant-interaction style (host and tissue specificity, symptom appearance) implies the importance of lineage-specific adaptations [6,7]. Combined efforts in genomic and transcriptomic research have provided key insights into *Colletotrichum* fungi evolution. For instance, compared with other fungi, *Colletotrichum* genomes are markedly rich with pathogenicity-related genes including PCWDEs, proteases, SM biosynthetic enzymes, secreted effectors [3,7]. During pathogenesis, these genes express dynamically to fulfill stage-specific pathogenic functions [3, 13]. Moreover, the gain and loss of PCWDE protein families have been indicated to be important in shaping their host specificities [15,16].

**Fig 8:**
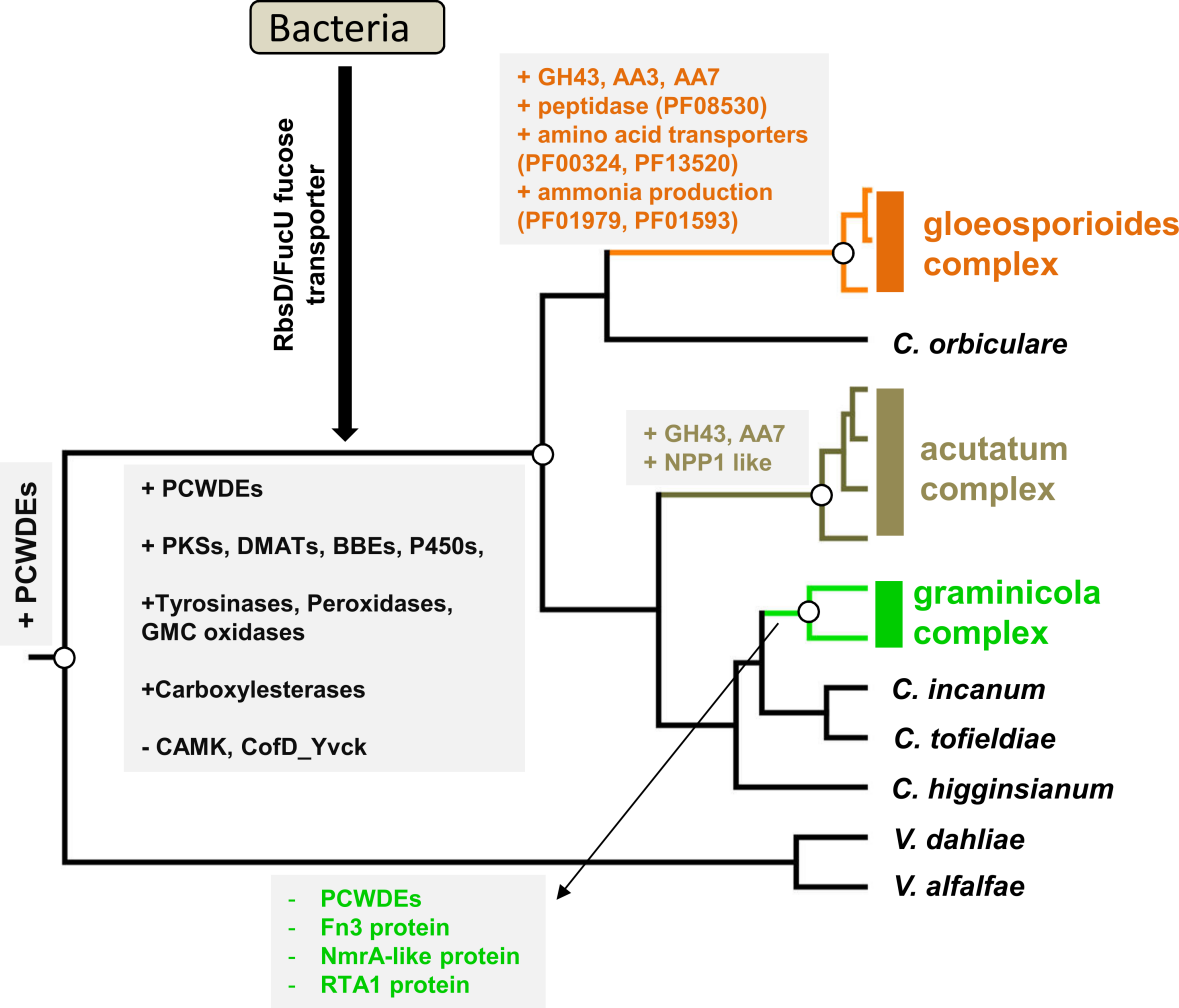
A summary representation of the important evolutionary events of *Colletotrichum* gene families.

In this study, we systematically compared the gene content variation across 13 *Colletotrichum* and 11 non-*Colletotrichum* genomes. Pathogenicity-related genes were annotated, classified, and compared; in addition, marked expansion/contraction events at key phylogenetic nodes were identified based on CAFE analysis. These results provided a global evolutionary picture of *Colletotrichum* gene families (summarized in Fig 8).

### Evolutionary dynamics of virulence-related gene families at the *Colletotrichum* MRCA

A range of gene families showed very strong expansions at the *Colletotrichum* MRCA. These include berberine bridge enzyme and PF11807 related to SM biosynthesis; type II peroxidase, tyrosinase and multicopper oxidase families related to oxidoreduction; carboxylesterase and tannase related to detoxification; OPT oligopeptide transporter and cytosine/purine permeases related to transport. Moreover, OrthoMCL analysis identified a range of core *Colletotrichum* genus-specific protein families with putative virulence roles including necrosis-induction (Hce2), signaling (CFEM), protein-oligosaccharide interactions (CVNH, WSC, PAN), and appressorium development (CAP22, CAS1), these genes are specific to *Colletotrichum* and conservatively present in all compared *Colletotrichum* genomes, and may thus be important for *Colletotrichum* infection. We have also identified three lineage-specific losses and one bacterial-derived horizontal transfer event at the *Colletotrichum* MRCA, demonstrating that lineage-specific gene loss and horizontal transfer have also contributed to *Colletotrichum* evolution.

*Colletotrichum* and *Verticillium* are related phytopathogens in the Glomerellales order, the former belongs to Glomerellaceae whereas the later belongs to Plectosphaerellaceae. Differing from *Colletotrichum* pathogens which mainly colonize leaves and fruits, *Verticillium* pathogens mainly colonize the plant root and vascular system. On the phylogenetic tree, the enrichment of pectinases was observed with both *Colletotrichum* and *Verticillium*, whereas many SM genes (e.g., synthetases, P450s, transporters), redox and detoxification-related enzymes are specifically enriched with *Colletotrichum*. Thus, these two categories of virulence factors appear to have different evolutionary histories although all being strongly expanded in *Colletotrichum*. The co-enrichment of pectin-degrading enzymes in *Colletotrichum* and *Verticillium* could be due to either single duplication prior to divergence or recent duplications related to independent adaptations. Plectosphaerellaceae family contains pathogenic genera such as *Plectospherella* and *Gibellulopsis* in addition to *Verticillium* [66], analyzing these genomes will be critical to understand PCWDE evolution in the Glomerellales.

### Evolutionary dynamics of virulence-related gene families among different *Colletotrichum* lineages

Among the 13 compared isolates, *C*. *sublineola* and *C*. *graminicola* specialize on monocot plants whereas other isolates specialize on dicot plants. In addition, species belonging to the acutatum complex and the gloeosporioides complex are more commonly observed to colonize fruits. Previous studies have reported a reduced set of pectin-degrading enzymes in *C*. *graminicola* and an elevated set of plant cell wall degrading enzymes in the acutatum and gloeosporioides complexes [3, 15, 16]. Our systemic CAFE analysis of gene family size evolution confirmed these results. More importantly, we identified a range of additional gene families showing gain or loss patterns relevant to such lineage-specific pathogenic adaptations.

*Colletotrichum* species are ‘alkaline’ fungi, accumulating high-level of ammonia both in culture and during plant infection, which is reportedly important for fungal infections [3, 67]. Two protein families with putative amidohydrolase activities (PF01979, PF04909) were significantly expanded at the *Colletotrichum* MRCA. Moreover, these two families together with four additional families related to ammonia production were further expanded at the gloeosporioides complex MRCA, suggesting a stepwise improvement in ammonia-producing potential. In the gloeosporioides complex, a deamination-related glutamate dehydrogenase plays significant roles in ammonia production, and the enzymatic activity requires amino acids as substrate [67]. In this study, the flavin containing amine oxidoreductase family (PF01593), which catalyzes ammonia production by oxidizing monoamines and polyamines [68], showed strong expansion in the gloeosporioides complex. *Colletotrichum* species belonging to the gloeosporioides complex are well-known fruit-infecting pathogens, their host fruit tissues are generally acidic in pH and these pathogens can modulate host local pH to promote infection [67, 69]. The expansion of flavin containing amine oxidoreductase might thus represent a virulence-relevant adaptation strategy in terms of pH regulation.

Another important protein family related to lineage-specific pathogenic adaptation is RTA1, which showed strong size reduction in the monocot-specializing graminicola complex. The family size was on-average one half that of other *Colletotrichum* species. As limited information is known regarding the biological functions of RTA1 proteins in filamentous fungi, it is difficult to interpret the significance of its reduction. Yet, in yeast, RTA1 overexpression confers drug or toxin tolerance [65], indicating a potential function of detoxifying monocot-relevant defense compounds.

### The evolution of lineage-specific genes in *C*. *fructicola*

*C*. *fructicola* has a broad host range, however pathogenicity test indicates that this species might encompass individual host-limited forms [19]. In this study, we compared the genomes of 1104–7 and Nara_gc5, two *C*. *fructicola* isolates derived from different hosts. The two genome assemblies were similar in size (57.1 Mb vs 55.6 Mb), shared 98.7% nucleotide identity in the alignment regions, up to 52.9 Mb of the 1104–7 genome were in > 10 kb alignment blocks when comparing with Nara_gc5. Thus, from a whole genome perspective, 1104–7 and Nara_gc5 were highly similar. By applying the same gene prediction pipeline to the 1104–7 and Nara_gc5 assemblies, their gene content variations could be compared in a non-biased manner. Interestingly, although similar total gene models were predicted (17,827 vs 17,844), OrthoMCL clustering identified approximately 1,000 isolate-specific genes in each genome, many of which may represent true genes based on the finding that over 60% of these genes had significant NCBI BlastP hits and that approximately 65% of the genes in 1104–7 had RNA-seq support.

Many fungal plant pathogen genomes can be classified into conserved core regions and plastic variable regions [70–72]. A plastic and fast-evolving subgenome is beneficial for deriving new host adaptations by elevating intraspecific diversification [70–72]. Although the biological traits of 1104–7 and Nara_gc5 have not been compared side by side, it is likely that the observed gene content variations are related to local adaptations. A plausible explanation for the high-degree of genome nucleotide identity and the existence of large numbers of isolate-specific genes would be that the *C*. *fructicola* genome encompasses subregions evolving at different speeds. To further dissect the intraspecific genomic variation among the two *C*. *fructicola* isolates, we identified and examined the evolutionary characteristics of genes located in lineage-specific (LS) regions in both genomes. With a length criterion of 10 kb, 0.62 Mb LS regions were identified in 1104–7 whereas 0.33 Mb LS regions were identified in Nara_gc5. Genes located within the LS regions are highly dynamic from an evolutionary perspective. Based on Blast queries, an elevated proportion of genes have no hit or are more closely related to non-*Colletotrichum* sequences than to *Colletotrichum* sequences. Moreover, two gene clusters showing strong signatures of fungus-to-fungus horizontal transfer were identified from the 1104–7 LS genomic regions. The putative functions of genes on the two clusters include serine protease, hemolysin-III protein known to function in membrane toxicity [73], as well as enzymes catalyzing secondary metabolite biosynthesis, all of which are virulence-related. While the host specificities of the two *C*. *fructicola* isolates have not been directly compared, the presence of virulence-related genes at the plastic subgenomic regions do support lineage-specific adaptations. In the *C*. *gloeosporioides* species complex, a strain-wide presence-absence polymorphism pattern of conditionally dispensable chromosomes (CDCs) has been observed [74], CDCs can transfer among strains even though direct evidence supporting their roles in pathogenicity transfer is lacking [75, 76]. In the future, determining whether the *C*. *fructicola* LS genomic DNAs identified in this study represent CDC and are virulence related will be of significant interest.

## Supporting information

S1 Fig Maximum likelihood (ML) based phylogenies of genes in the 1104–7 HGT1 and HGT2 clusters. Maximum likelihood (ML) based phylogenies of genes in the 1104–7 HGT1 and HGT2 clusters. For each gene (red color), best non-Colletotrichum BlastP hits (black nodes) and best Colletotrichum hits (green nodes) were retrieved from NCBI nr database, aligned for ML tree construction in RAxML 8.1.1. The best amino acid substitution models (shown for each tree) were identified with ProtTest3. Bootstrap values (based on 1,000 replicates) are indicated for major nodes.
(PDF)

S2 Fig Carbohydrate-active enzyme (CAZY) content variation among compared genomes. Carbohydrate-active enzyme (CAZY) content variation among compared genomes. GH, glycoside hydrolase; GT, glycoside transferase; PL, polysaccharide lyases; CE, carbohydrate esterase; CBM, carbohydrate-binding modules; AA, auxiliary activities.
(PDF)

S3 Fig Variation of secreted proteases among compared genomes. A, aspartic type; M, metallo type; S, serine type.
(PDF)

S4 Fig Variation of secondary metabolite synthetases among compared genomes. DMAT, dimethylallyl tryptophan transferase; NRPS, nonribosomal peptide synthase; PKS, polyketide synthase; TS, terpene synthase; HYBRID, NRPS-PKS hybrid.
(PDF)

S5 Fig Variation of cytochrome P450s among compared genomes. (PDF)

S6 Fig Variation of transporter genes among compared genomes. (PDF)

S7 Fig Variation of small secreted protein (SSP) content among compared genomes. SSPs are defined as proteins containing predicted secretion signals and being less than 300 aa. CSSPs, cysteine-rich SSPs (cysteine% > 3%); NCSSPs, non cysteine-rich SSPs (cysteine% ≤ 3%).
(PDF)

S1 File The gene annotations and prdicted protein sequences of the *C*. *fructicola* 1104–7 genome. (RAR)

S2 File Table A to E.
(XLSX)
