# Supplementary figures and images for: Pathogenic adaptations of Colletotrichum fungi revealed by genome wide gene family evolutionary analyses
Source: PLoS One. 2018 Apr 24;13(4):e0196303. doi: 10.1371/journal.pone.0196303 (PMC5915685; doi:10.1371/journal.pone.0196303)

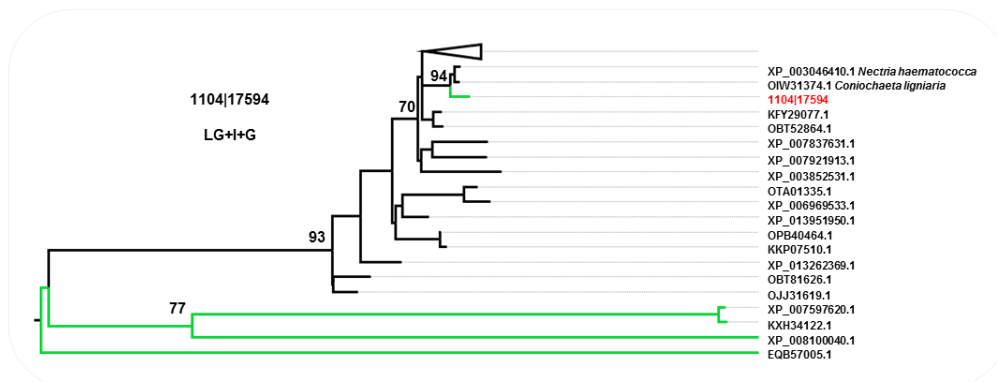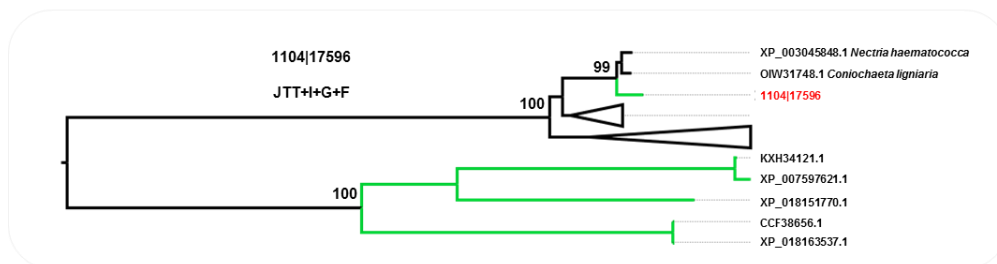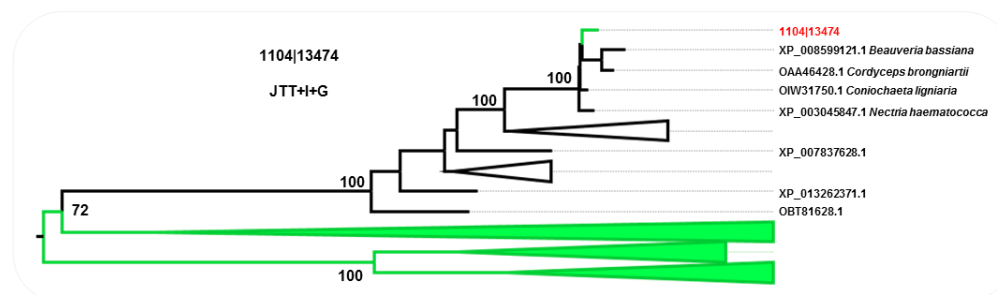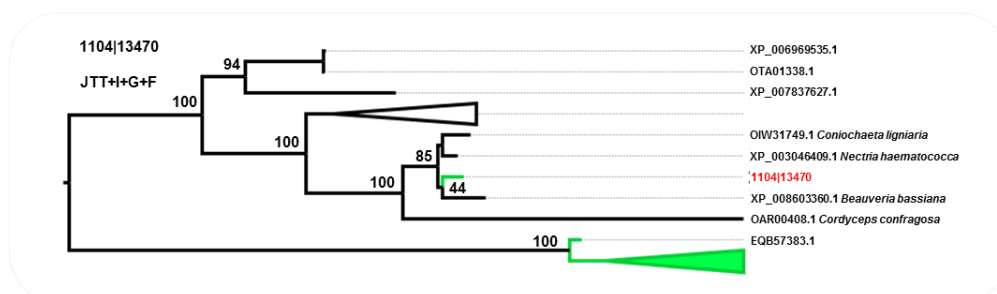

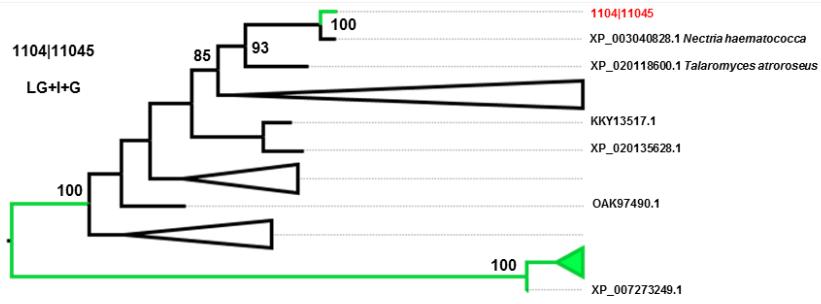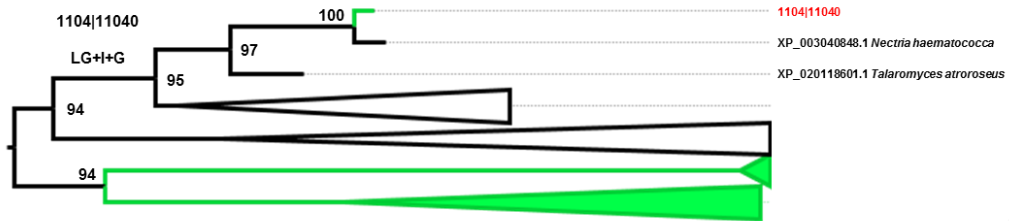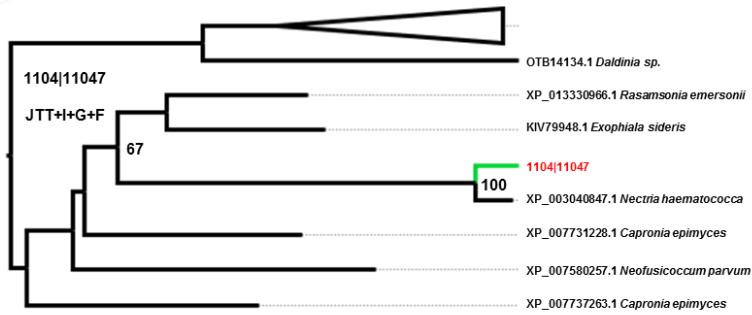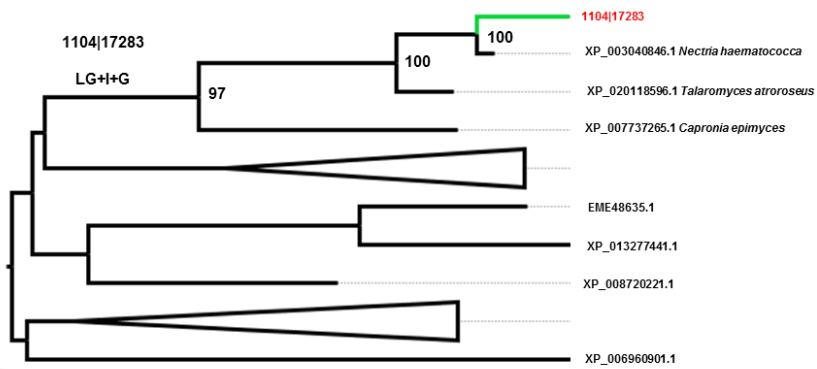

Supplement: S1 Fig — Maximum likelihood (ML) based phylogenies of genes in the 1104–7 HGT1 and HGT2 clusters. For each gene (red color), best non-Colletotrichum BlastP hits (black nodes) and best Colletotrichum hits (green nodes) were retrieved from NCBI nr database, aligned for ML tree construction in RAxML 8.1.1. The best amino acid substitution models (shown for each tree) were identified with ProtTest3. Bootstrap values (based on 1,000 replicates) are indicated for major nodes. (PDF) [file pone.0196303.s001.pdf]

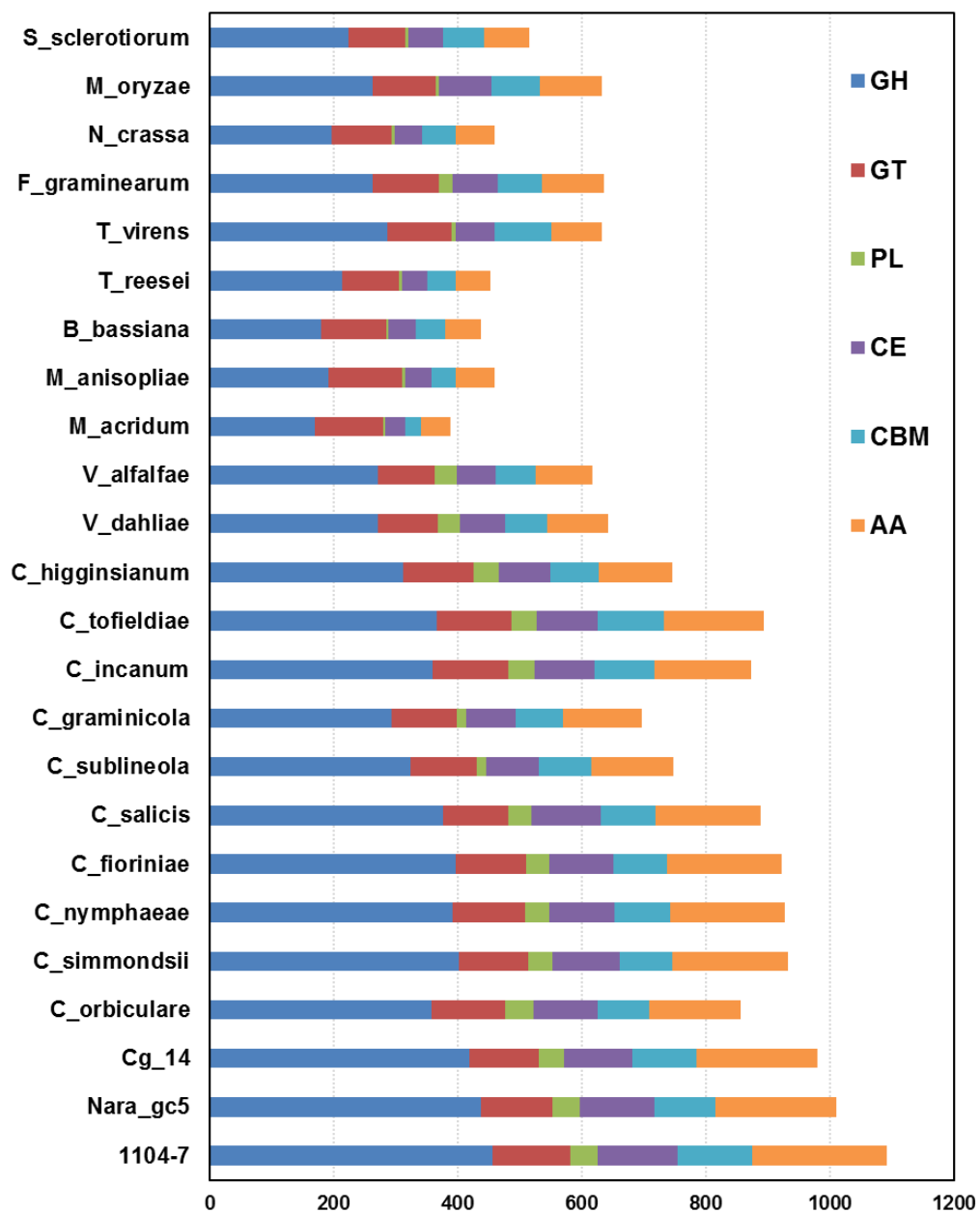

Supplement: S2 Fig — Carbohydrate-active enzyme (CAZY) content variation among compared genomes. GH, glycoside hydrolase; GT, glycoside transferase; PL, polysaccharide lyases; CE, carbohydrate esterase; CBM, carbohydrate-binding modules; AA, auxiliary activities. (PDF) [file pone.0196303.s002.pdf]

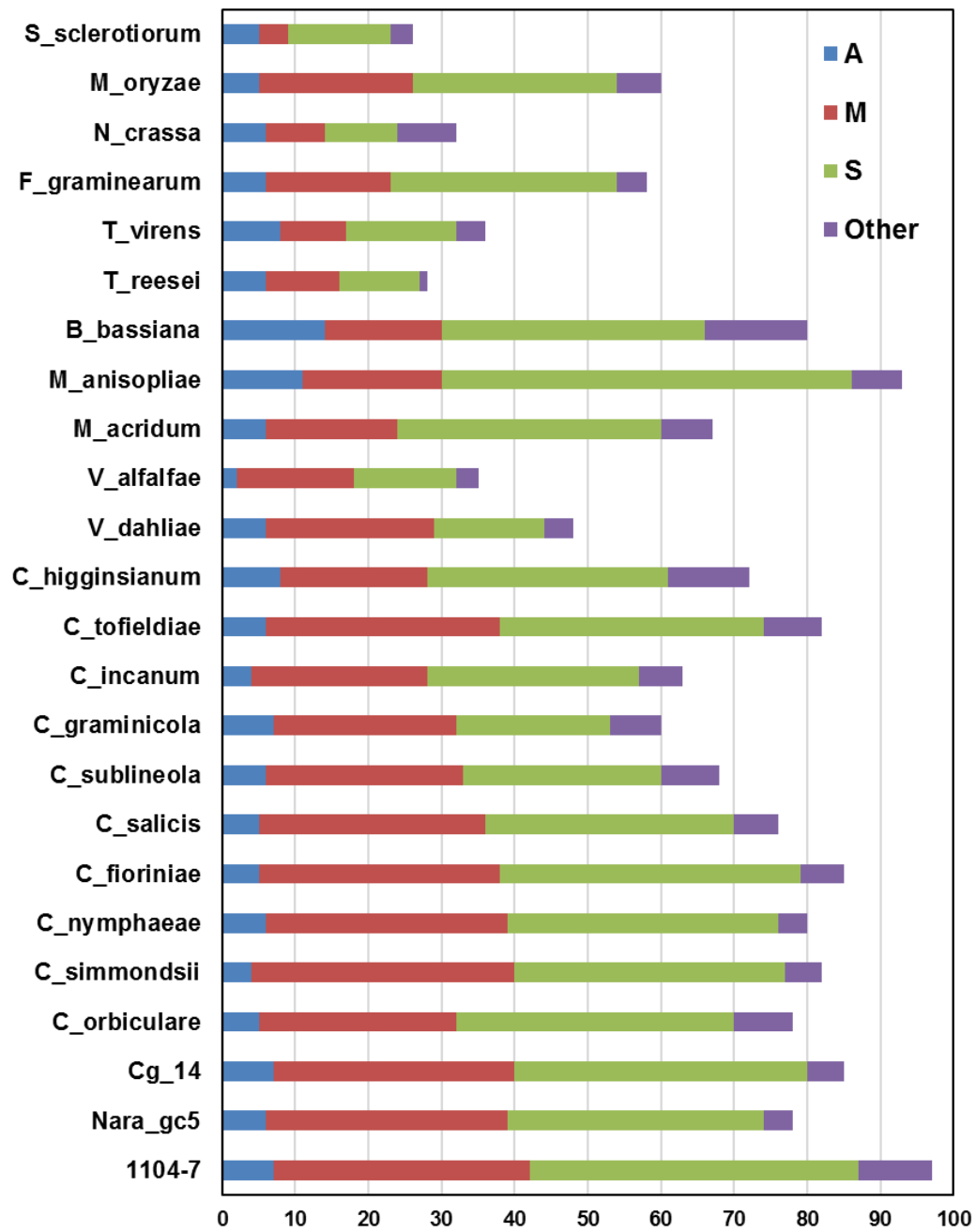

Supplement: S3 Fig — A, aspartic type; M, metallo type; S, serine type. (PDF) [file pone.0196303.s003.pdf]

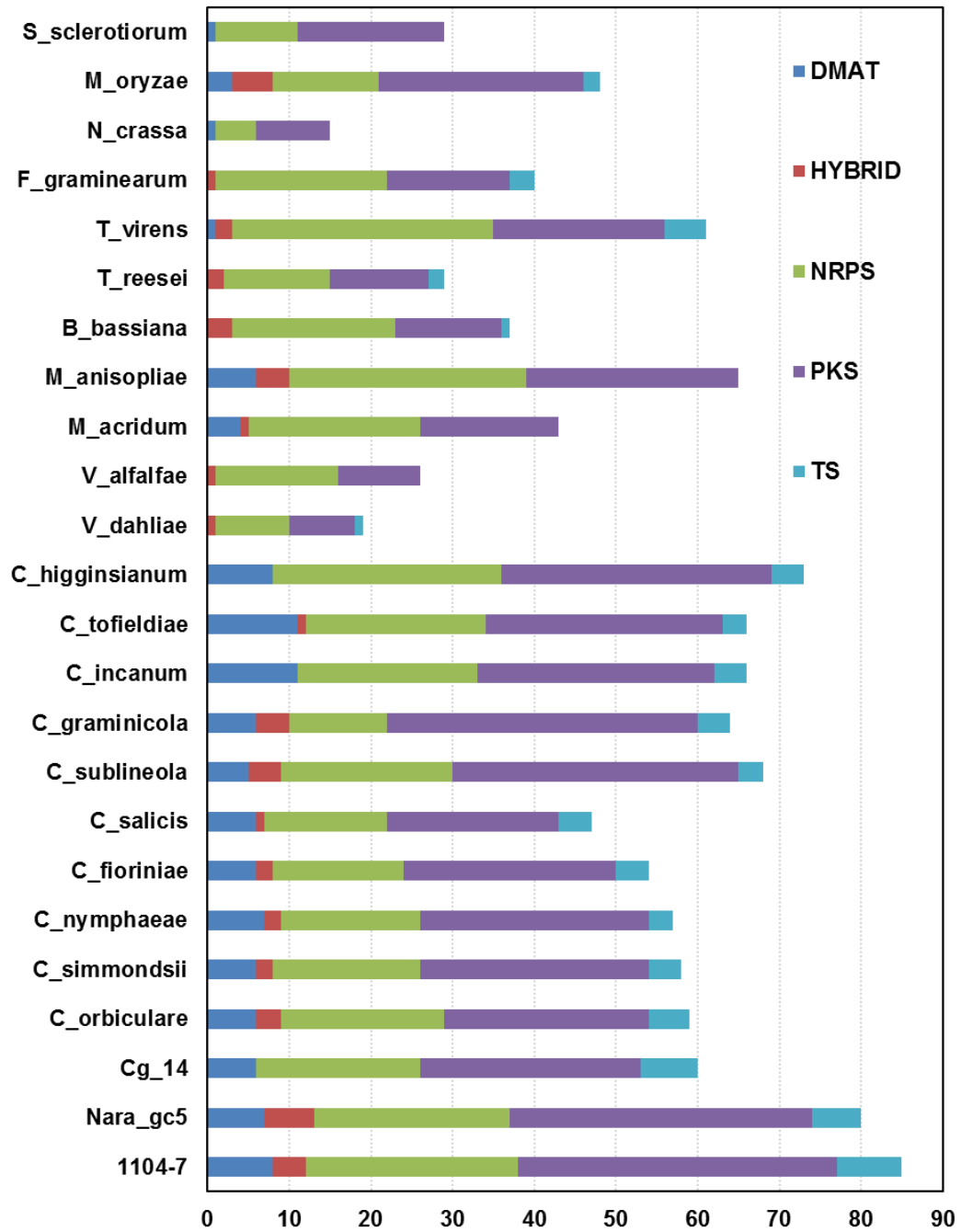

Supplement: S4 Fig — DMAT, dimethylallyl tryptophan transferase; NRPS, nonribosomal peptide synthase; PKS, polyketide synthase; TS, terpene synthase; HYBRID, NRPS-PKS hybrid. (PDF) [file pone.0196303.s004.pdf]

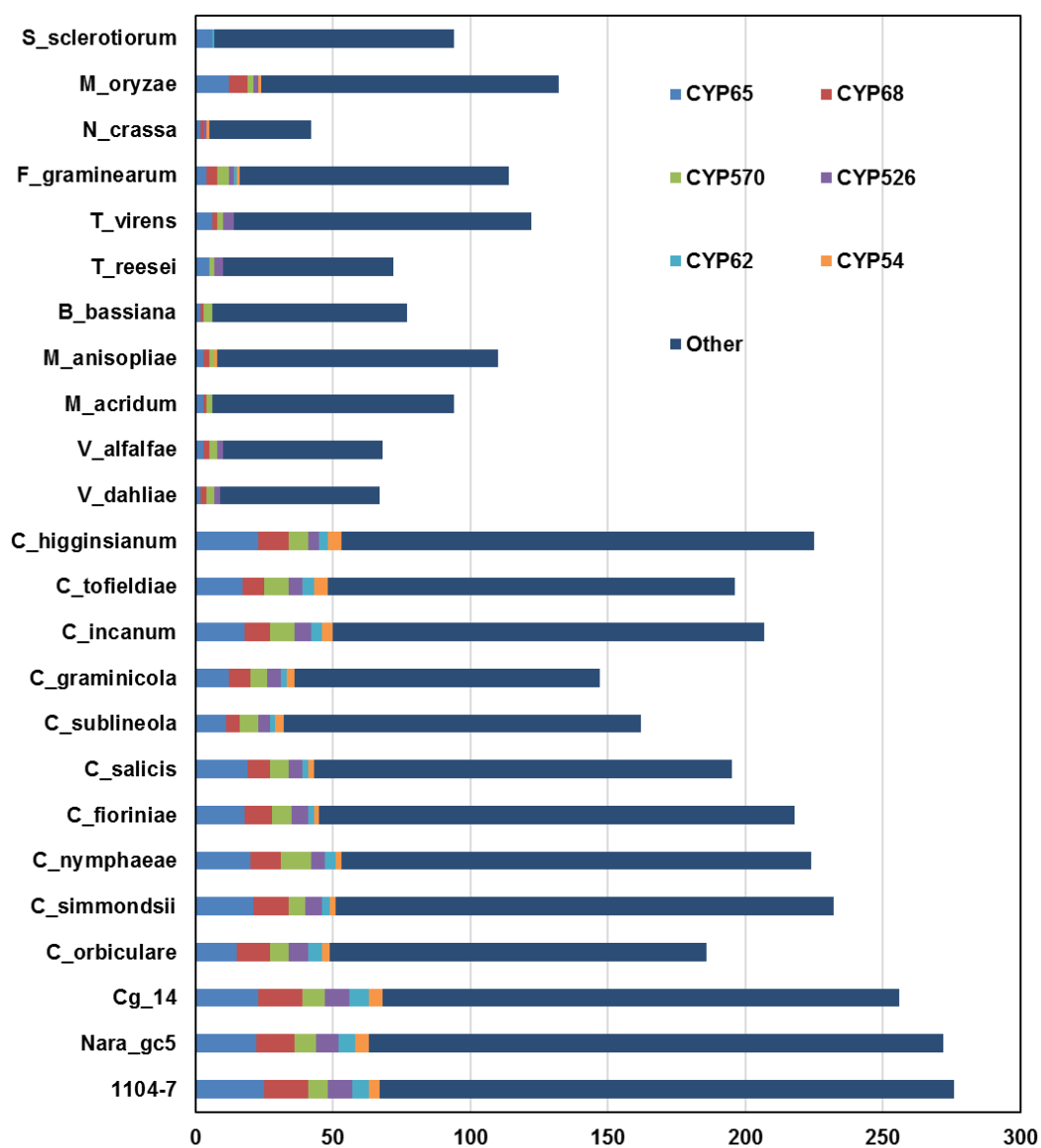

Supplement: S5 Fig — (PDF) [file pone.0196303.s005.pdf]

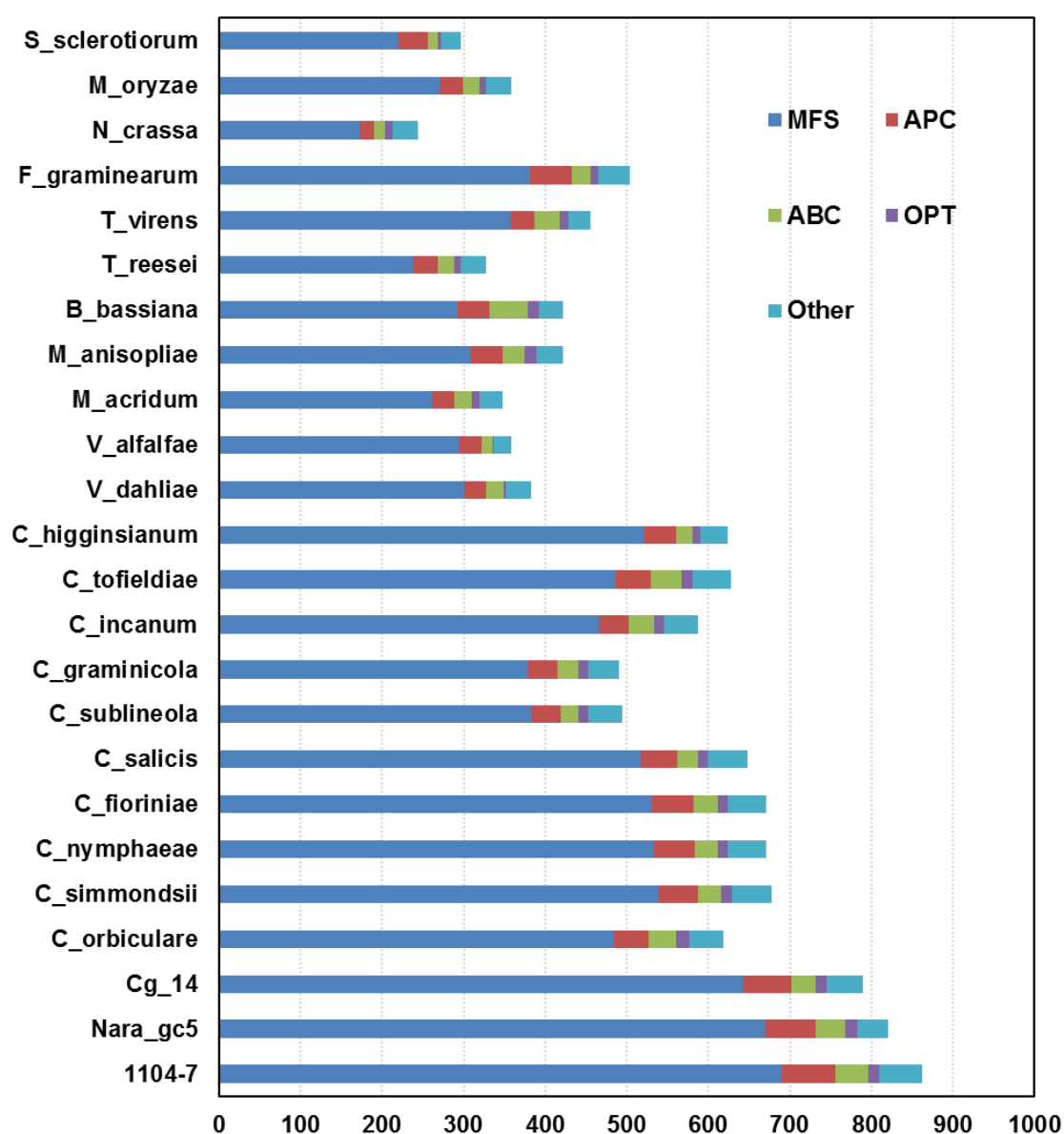

Supplement: S6 Fig — (PDF) [file pone.0196303.s006.pdf]

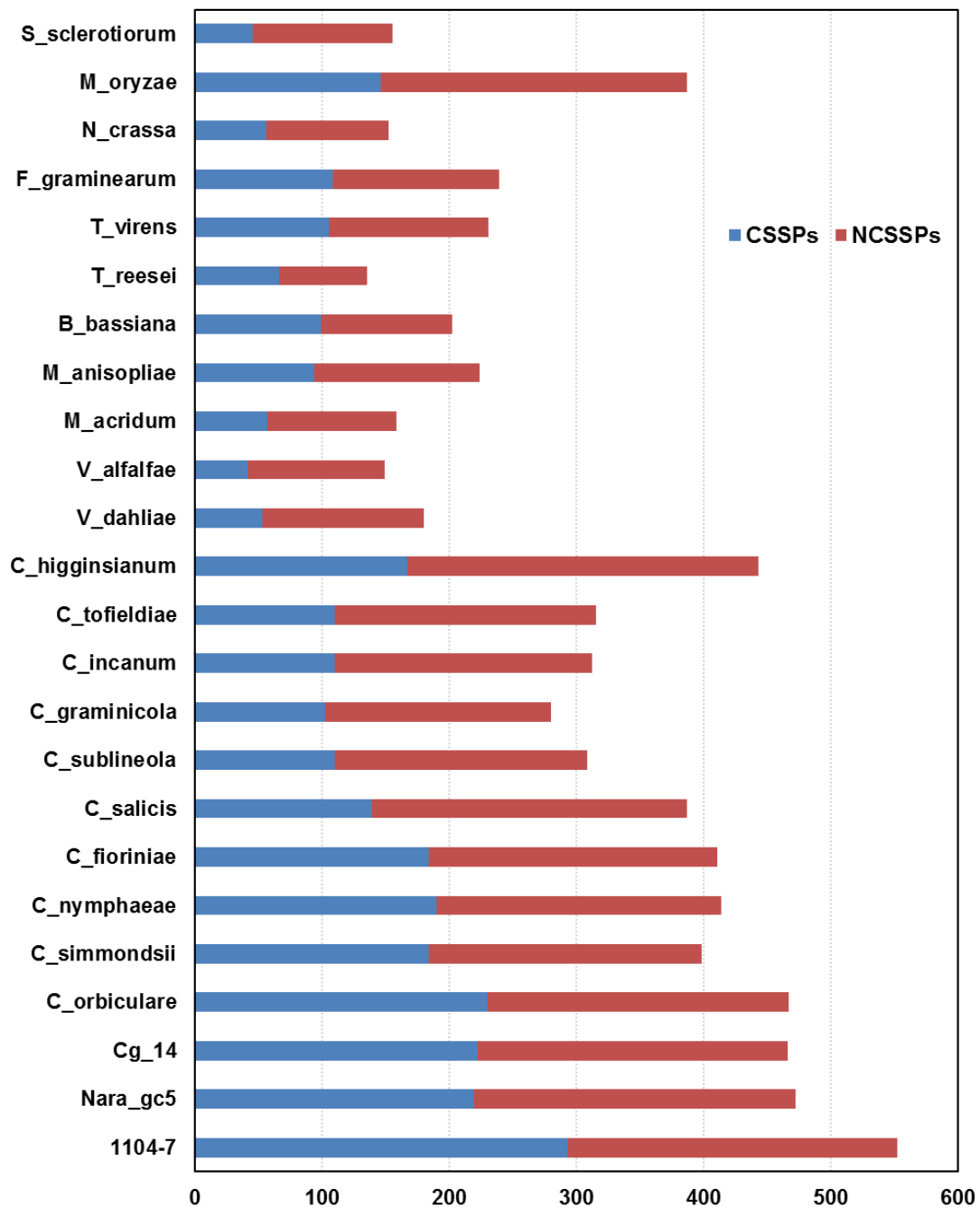

Supplement: S7 Fig — SSPs are defined as proteins containing predicted secretion signals and being less than 300 aa. CSSPs, cysteine-rich SSPs (cysteine% > 3%); NCSSPs, non cysteine-rich SSPs (cysteine% ≤ 3%). (PDF) [file pone.0196303.s007.pdf]
